# Fluorescent GLP1R/GIPR dual agonist probes reveal cell targets in the pancreas and brain

**DOI:** 10.1038/s42255-025-01342-6

**Published:** 2025-08-19

**Authors:** Anne de Bray, Anna G. Roberts, Sarah Armour, Jason Tong, Christiane Huhn, Blaise Gatin-Fraudet, Kilian Roßmann, Ali H. Shilleh, Wanqing Jiang, Natalie S. Figueredo Burgos, James P. P. Trott, Katrina Viloria, Daniela Nasteska, Abigail Pearce, Satsuki Miyazaki, Jeremy W. Tomlinson, Dylan M. Owen, Daniel J. Nieves, Julia Ast, Malgorzata Cyranka, Alexey Epanchintsev, Carina Ämmälä, Frank Reimann, Tolga Soykan, Graham Ladds, Alice E. Adriaenssens, Stefan Trapp, Ben Jones, Johannes Broichhagen, David J. Hodson

**Affiliations:** 1 Department of Metabolism and Systems Science, College of Medicine and Health, University of Birmingham, Birmingham, UK; 2 Oxford Centre for Diabetes, Endocrinology and Metabolism (OCDEM), NIHR Oxford Biomedical Research Centre, Churchill Hospital, Radcliffe Department of Medicine, University of Oxford, Oxford, UK; 3 Centre for Cardiovascular and Metabolic Neuroscience, Department of Neuroscience, Physiology & Pharmacology, UCL, London, UK; 4 Leibniz-Forschungsinstitut für Molekulare Pharmakologie, Berlin, Germany; 5 Department of Pharmacology, University of Cambridge, Cambridge, UK; 6 Division of Stem Cell Regulation Research, Center for Medical Research and Education, Osaka University Graduate School of Medicine, Suita, Osaka, Japan; 7 Department of Immunology and Immunotherapy, School of Infection, Inflammation and Immunology, College of Medicine and Health, University of Birmingham, Birmingham, UK; 8 Centre of Membrane Proteins and Receptors, University of Birmingham, Birmingham, UK; 9 School of Mathematics, University of Birmingham, Birmingham, UK; 10 Novo Nordisk Research Centre Oxford, Innovation Building, Oxford, UK; 11 Institute of Metabolic Science-Metabolic Research Laboratories & MRC-Metabolic Diseases Unit, University of Cambridge, Cambridge, UK; 12 Section of Endocrinology and Investigative Medicine, Imperial College London, London, UK

**Keywords:** Type 2 diabetes, Obesity, Receptor pharmacology, Super-resolution microscopy

## Abstract

Dual agonists targeting glucagon-like peptide-1 receptor (GLP1R) and glucose-dependent insulinotropic polypeptide receptor (GIPR) are breakthrough treatments for patients with type 2 diabetes and obesity. Compared to GLP1R agonists, dual agonists show superior efficacy for glucose lowering and weight reduction. However, delineation of dual agonist cell targets remains challenging. Here, we develop and test daLUXendin and daLUXendin+, non-lipidated and lipidated fluorescent GLP1R/GIPR dual agonist probes, and use them to visualize cellular targets. daLUXendins are potent GLP1R/GIPR dual agonists that advantageously show less functional selectivity for mouse GLP1R over mouse GIPR. daLUXendins label rodent and human pancreatic islet cells, with a signal intensity of β cells > α cells = δ cells. Systemic administration of daLUXendin strongly labels GLP1R^+^ and GIPR^+^ neurons in circumventricular organs characterized by an incomplete blood–brain barrier but does not penetrate the brain beyond labelling seen with single (ant)agonists. At the single-molecule level, daLUXendin targets endogenous GLP1R–GIPR nanodomains, which differ in organization and composition from those targeted by a single agonist. daLUXendins reveal dual agonist targets in the pancreas and brain and exclude a role for brain penetration in determining the superior efficacy of dual agonists, shedding new light on different modes of action of dual agonists versus single agonists.

## Abstract (web summary)

A series of fluorescent probes is designed that act as dual agonists on both GLP1 and GIP receptors, and are used to image receptor binding activities in tissues and animals.

## Main

Glucagon-like peptide-1 (GLP1) and glucose-dependent insulinotropic polypeptide (GIP) potently amplify insulin secretion and influence satiety to reduce food intake^1–3^. Stabilized GLP1R agonists were approved for the treatment of type 2 diabetes in 2008 (ref. ^4^) and at higher doses for overweight and obesity in 2017 and 2018 (ref. ^5^). Less attention has been paid to the GIP axis, as multiple studies showed that either genetic ablation or pharmacological blockade of GIPR protects against body weight gain^6^, and that GIPR agonism has minimal effects on glycemia in patients with type 2 diabetes^7^, potentially owing to GIPR downregulation^8^. However, dual agonists such as tirzepatide combine elements of GLP1R and GIPR agonism, such that the full benefits of both hormone axes are realized^9^.

Although the pharmacological mechanisms underlying dual GLP1R/GIPR agonist efficacy are well established^10–12^, less is known about the cellular substrates that underlie actions on pancreatic hormone secretion as well as food intake. GLP1R and GIPR are low-abundance cell surface proteins, and their instability in solution makes it difficult to raise antibodies against specific epitopes, rendering their immunohistochemical visualization challenging^13^. Specific antibodies exist for GLP1R, but no reliable commercial antibody exists yet for the detection of GIPR in primary cells and tissues^13^. Compared to antibodies, GLP1R-Cre and GIPR-Cre reporter mice are sensitive, but only identify cells that express(ed) GLP1R/GIPR transcripts^14,15^. Finally, animals harbouring a self-labelling enzyme on the GLP1R amino terminus allow facile visualization using a range of fluorophores, although this model is restricted to rodents^16^. No approach to date provides information on GLP1Rs and GIPRs specifically accessed or bound by a dual agonist.

To circumvent these issues, we previously developed LUXendins and sGIPs^17–19^, which are specific probes against GLP1R and GIPR, respectively. Using these probes, we confirmed that GLP1R is largely a β cell-specific protein marker^18,20^, whereas GIPR is expressed in both β cells and α cells within the islet^17^. However, we were unable to make any inferences about dual agonist binding sites within the islet and brain, as GLP1R and GIPR probes might target different receptor (sub)populations. Dual agonism might also influence GIPR/GLP1R localization, expression and access through effects in addition to those mediated by GLP1R and/or GIPR (ant)agonism alone; for example, release of paracrine mediators and functional selectivity^21–23^. Although fluorescent tirzepatide has been reported^12^, the probe was not pharmacologically validated for its specificity at GLP1R/GIPR.

### Design of daLUXendin544 and daLUXendin660

**Fig. 1:**
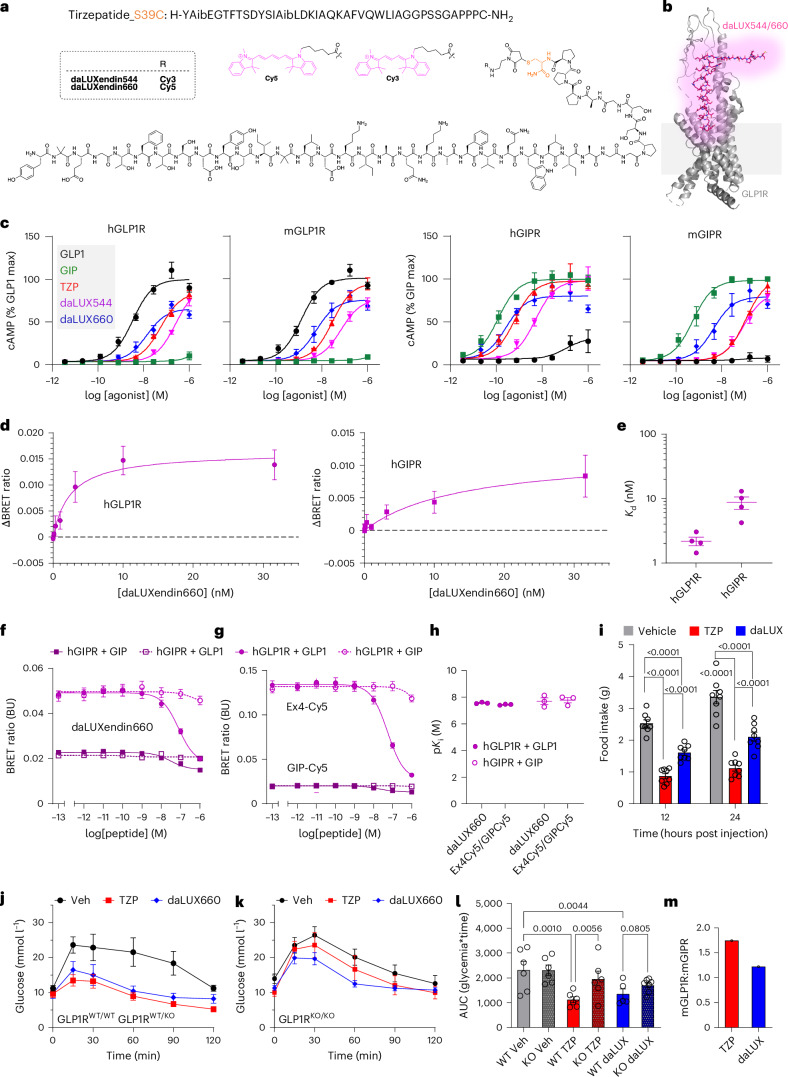
Development and characterization of daLUXendin544 and daLUXendin660. **a**,**b**, Tirzepatide, modified at the C-terminus with S39C to allow maleimide conjugation with either Cy3 or Cy5 (model obtained from cryo-electron microscopic structure of non-acyl tirzepatide bound to activated GLP1R in complex with G_s_) (PDB 7VBI)^26^. **c**, cAMP responses to GLP1, GIP, tirzepatide (TZP), daLUXendin544 (daLUX544) and daLUXendin660 (daLUX660) in AD293 cells expressing either hGLP1R, mGLP1R, hGIPR or mGIPR (*n* = 4 independent repeats). **d**,**e**, Binding affinity of daLUXendin660 against hGLP1R and hGIPR (**d**), with *K*_d_ values (**e**) in a separate bar graph (one-site fit specific binding) (*n* = 3 independent repeats). **f**,**g**, Competition binding for daLUXendin660 (**f**) and either Ex4-Cy5 or GIP-Cy5 (**g**) at hGLP1R and hGIPR versus GLP1 and GIP (one-site fit *K*_i_) (*n* = 3 independent repeats). **h**, p*K*_i_ values for daLUXendin660, Ex4-Cy5 and GIP-Cy5 at hGLP1R and hGIPR versus GLP1 and GIP (*n* = 3 independent repeats). **i**, Mice were fasted 3 h before the onset of the dark phase. At dark onset, mice were injected subcutaneously with either vehicle, daLUXendin660 (daLUX; 10 nmol kg^−1^) or tirzepatide (10 nmol kg^−1^), and then food was returned. Graph showing cumulative food intake 12 h and 24 h post injection (*n* = 8 mice per investigated state) (repeated-measures two-way ANOVA with Šídák’s multiple comparisons test). **j**, Tirzepatide and daLUXendin660 similarly lower glucose in GLP1R^WT/WT^ and GLP1R^WT/KO^ mice during glucose tolerance testing (*n* = at least five mice per investigated state). **k**,**l**, Efficacy of tirzepatide and daLUXendin660 is reduced in GLP1R^KO/KO^ mice (**k**), shown also by area under the curve (AUC) (**l**) (*n* = at least five mice per investigated state) (one-way ANOVA with two-stage linear step-up procedure of Benjamini, Krieger and Yekutieli). **m**, mGLP1R:mGIPR selectivity is lower for daLUXendin660 versus tirzepatide (selectivity is calculated using the mean AUC ratio from at least five GLP1R^WT/WT^ and five GLP1R^KO/KO^ mice). Bar and line graphs show means; error bars, s.e.m. ***P < 0.001. Exact *P* values are displayed on each graph. Source data

Structure–activity relationships and cryo-electron microscopy show that the binding affinity and efficacy of GLP1R agonists, GIPR agonists and dual agonists are determined by amino acids closer to the N-terminus^24–27^. We thus reasoned that carboxy-terminal functionalization with a fluorophore would be well tolerated, especially because this end is not commonly resolved in structural interpretations. Given that tirzepatide possesses a C-terminal serine, we decided to generate an S39C mutant, allowing the late-stage addition of a fluorophore (Fig. 1a,b). daLUXendin544 and daLUXendin660 were obtained using cysteine-maleimide ‘click’ chemistry to install Cy3 and Cy5 fluorophores, respectively, in yields of ~50%. *iso*-butyric amino acid was incorporated into the structure instead of alanine at position two to confer degradation resistance against DPP-IV^28^ (Fig. 1a,b). Spectral properties, compound purity and compound characterization are shown in Supplementary Fig. 1 and Supplementary Note 1.

### Pharmacology of daLUXendin544 and daLUXendin660

AD293 cells transfected with either human GLP1R (hGLP1R), mouse GLP1R (mGLP1R), human GIPR (hGIPR) or mouse GIPR (mGIPR) were used to determine cAMP signalling potency of daLUXendin544 and daLUXendin660 versus unmodified (acylated) tirzepatide, GIP and GLP1. At both hGLP1R and mGLP1R, tirzepatide and its fluorescent analogues showed reduced potency compared to GLP1, with the potency rank order favouring daLUXendin660 > tirzepatide > daLUXendin544 (Fig. 1c) (Extended Data Table 1). As previously reported for tirzepatide^11^, there were species differences for GIPR signalling with each of the dual agonist ligands tested, with all showing a larger loss of potency at the mGIPR than at the hGIPR relative to native hGIP (Fig. 1c) (Extended Data Table 1 and Supplementary Table 1). Of note, even though daLUXendin544 showed globally reduced potency compared to daLUXendin660, half-maximum effective concentration (EC_50_) ratios suggested that both ligands show similar GIPR:GLP1R selectivity of ~2:1 (that is, favouring GIPR) for human receptors and ~1:2 (that is, favouring GLP1R) for mouse receptors. By comparison, tirzepatide showed GIPR:GLP1R selectivity of ~3:1 for human receptors and ~1:8 for mouse receptors in our assays.

daLUXendin660 showed nM binding affinity at both hGLP1R and hGIPR, although assessment of the daLUXendin544 dissociation constant (*K*_d_) was precluded by interference with the BRET signal (Fig. 1d,e) (Supplementary Table 2). Competition assays showed that binding affinity (p*K*_i_) for hGLP1/hGLP1R and hGIP/hGIPR was similar when using daLUXendin660 as a probe compared to Ex4-Cy5 and GIP-Cy5, further confirming strong receptor binding (Fig. 1f–h) (Supplementary Table 3).

Therefore, based upon cAMP assays and binding affinity, daLUXendin544 and daLUXendin660 are potent GLP1R/GIPR dual agonists that advantageously show approximately fourfold less selectivity for mouse GLP1R over GIPR compared to tirzepatide.

### daLUXendin660 shows less selectivity for GLP1R:GIPR in vivo

To establish whether in vitro pharmacology is mirrored by in vivo efficacy, mice were dosed with either tirzepatide or daLUXendin660 at 10 nmol kg^−1^ to maximally engage mGLP1R and mGIPR^11,29^. At this dose, daLUXendin660 and tirzepatide both reduced food intake during the dark phase and over a cumulative 24 h period. At 12 h and 24 h post injection, tirzepatide demonstrated ~40% greater efficacy in driving anorexia, presumably because of its acylation and plasma binding (Fig. 1i). Likewise, both tirzepatide and daLUXendin660 exerted substantial glucose-lowering effects in Glp1r^WT/WT^ and Glp1r^WT/KO^ mice, with similar area under the curves (Fig. 1j–l). Both ligands partially maintained their efficacy in Glp1r^KO/KO^ mice, reflecting continued engagement of mGIPR at doses >1–3 nmol kg^−1^ (Fig. 1j–l). However, Glp1r knockout was less effective in daLUXendin660-treated versus tirzepatide-treated mice, demonstrating increased mGIPR dependence for daLUXendin660, as expected from in vitro functional selectivity data (Fig. 1j–m). Results should, however, be interpreted in light of increased mGIPR sensitivity or expression in Glp1r^KO/KO^ mice^11^. Together, these data confirm that daLUXendin660 and tirzepatide both lower blood glucose and reduce food intake.

### Cell labelling with daLUXendin544 and daLUXendin660

**Extended Data Fig. 1:**
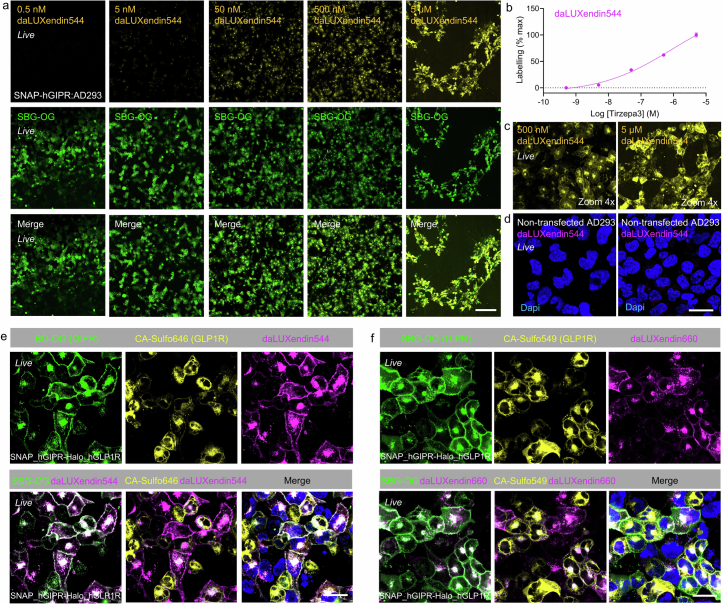
Validation of daLUXendin544 and daLUXendin660 labelling in cellulo. **a, b)** daLUXendin544 labels SNAP_hGIPR:AD293 cells in a concentration-dependent manner (SBG-OG; cell impermeable SNAP label), shown by representative images (**a**), and line graph (**b**) (n = at least 7 wells, 2 independent repeats). **c)** 500 nM daLUXendin544 brightly labels SNAP_hGIPR:AD293 cells without signs of impaired morphology (n = at least 7 wells, 2 independent repeats). **d)** daLUXendin544 and daLUXendin660 do not label non-transfected AD293 cells (n = at least 9 wells, at least 2 independent repeats). **e)** daLUXendin544 labels cells co-expressing hGIPR and hGLP1R bearing orthogonal SNAP- and Halo-tags, visualized using cell impermeable SBG-OG (GIPR) and CA-Sulfo646 (GLP1R) (n = 24 wells, 3 independent repeats). **f)** As for e), but daLUXendin660 (n = 19 wells, 3 independent repeats). Scale bar = 212.5 µm in a, scale bar = 33.7 µm in d-f. Source data

In line with the cAMP results, daLUXendin544 led to concentration-dependent labelling of SNAP-hGIPR:AD293 cells, overlapping with cell-impermeable SBG-Oregon Green (OG) SNAP label (Extended Data Fig. 1a,b)^30^. The brightest labelling was detected at 50 nM–5 µM, corresponding to the cAMP max (Extended Data Fig. 1a,b). The strongest and most specific membrane labelling, without evidence of altered cell morphology (Extended Data Fig. 1a–c), was observed with 500 nM daLUXendin544; hence, this concentration was selected for experiments in cell lines and tissue. No labelling was observed in non-transfected AD293 cells (Extended Data Fig. 1d). Both daLUXendin544 and daLUXendin660 showed colocalization with cells transfected with SNAP_GIPR and Halo_GLP1R, determined using orthogonal cell-impermeable SNAP (SBG-OG) and Halo (CA-Sulfo549 or CA-Sulfo646) labels^31^ (Extended Data Fig. 1e,f). Therefore, daLUXendin544 and daLUXendin660 are highly specific dual agonist probes suitable for cell labelling.

### daLUXendins label endogenous GIPR and GLP1R

**Extended Data Fig. 2:**
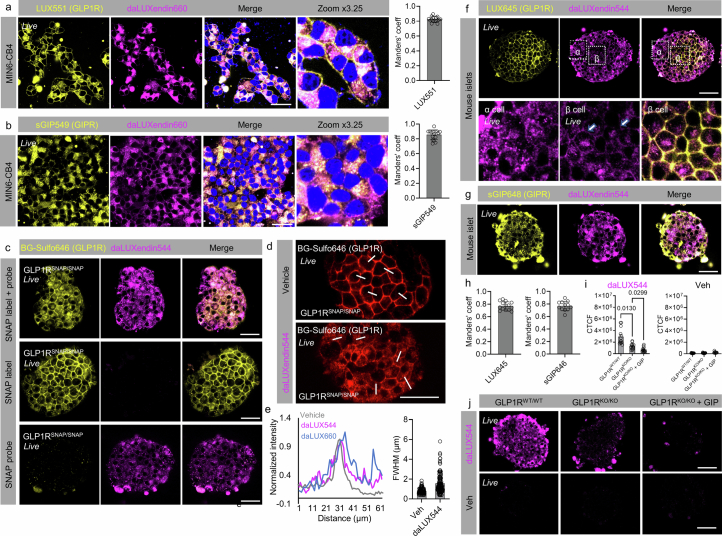
daLUXendin544 and daLUXendin660 specifically label endogenous GLP1R and GIPR. **a)** daLUXendin660 and LUXendin551 (LUX551; GLP1R probe) labelling co-localize in MIN6-CB4 beta cells, which endogenously express GLP1R (n = 16 images from 4 wells, 2 independent repeats). **b)** daLUXendin660 and sGIP549 (GIPR probe) co-localize in MIN6-CB4 beta cells, which endogenously express GIPR (n = 16 images from 4 wells, 2 independent repeats). **c)** daLUXendin544 labelling co-localizes with BG-Sulfo646 SNAP label in GLP1R^SNAP/SNAP^ mouse islets (n = 24 islets from 5 mice). **d, e)** daLUXendin544 labels membrane and cytoplasmic (that is, internalized) GLP1R pools in GLP1R^SNAP/SNAP^ mouse islets, labelled using the cell impermeable SNAP label, BG-Sulfo646, and shown using full width at half maximum (FWHM) (line placement used for FHWM calculation is shown) (n = at least 18 islets from at least 5 mice). **f)** daLUXendin544 engages distinct GLP1R/GIPR pools in α cells versus β cells, with more membrane labelling in β cells (arrows show membrane labelling) (n = 37 islets from 3 mice). **g)** daLUXendin544 and sGIP648 (GIPR) labelling co-localize in mouse islets (n = 37 islets from 3 mice). **h)** Analysis showing strong co-localization between daLUXendin544 and either GLP1R or GIPR, labelled using LUXendin645 (LUX645) and sGIP648, respectively (n = 43 islets from 2 mice). **i, j)** daLUXendin544 labelling is reduced in GLP1R^KO/KO^ islets, and further reduced in GLP1R^KO/KO^ islets + 1 µM GIP Aib2 (n = 37 islets from 2 mice) (Kruskal-Walllis test with two-stage linear step-up procedure of Benjamini, Krieger and Yekutieli). Scale bar = 33.7 µm in a and b; scale bar = 53 µm in c, d, f, g and j. No changes are seen in vehicle-treated islets. Bar graphs show mean ± SEM. Exact *p* values are displayed on each graph. Source data

MIN6-CB4 β cells were labelled with both daLUXendin660 and the specific GLP1R probe LUXendin551 (refs. ^18,19^). Demonstrating specificity for GLP1R, LUXendin551 signal colocalized with daLUXendin660 (Extended Data Fig. 2a), probably owing to labelling of different GLP1R pools (see below). However, not all daLUXendin660 signal colocalized with LUXendin551, presumably owing to GIPR labelling, as well as increased GLP1R internalization (Extended Data Fig. 2a). To investigate this possibility further, MIN6-CB4 β cells were incubated with daLUXendin660 and the specific GIPR probe sGIP549 (ref. ^17^) (Extended Data Fig. 2b). Demonstrating specificity for GIPR, sGIP549 signal colocalized with daLUXendin660, whereas not all daLUXendin660 signal colocalized with sGIP549, corresponding to GLP1R labelling (Extended Data Fig. 2b). Therefore, daLUXendin660 labels both endogenous GLP1R and GIPR in MIN6-CB4 β cells.

We next repeated studies in isolated mouse islets. To circumvent competition between the various probes at GLP1R/GIPR, we used GLP1R^SNAP/SNAP^ mice in which a SNAP-tag enzyme self-label is knocked into the extracellular domain of the endogenous GLP1R^16^, allowing GLP1R to be visualized without influencing orthosteric binding. Using this model, colocalization could be detected between the cell-impermeable SNAP label BG-Sulfo646 and daLUXendin544 (Extended Data Fig. 2c). We noticed that daLUXendin544 retained significant levels of GLP1R at the cell surface, as shown by the full width at half maximum (Extended Data Fig. 2d,e). By contrast, daLUXendin544 did not exhibit any detectable membrane labelling in α cells, presumably because they express little to no GLP1R transcript or protein (also used to identify α cells versus β cells) (Extended Data Fig. 2f,h)^15,18,20^, so α cell labelling is largely by GIPR. Using the GIPR probe sGIP648, daLUXendin544 was found to strongly label GIPR in mouse islets (Extended Data Fig. 2g,h), also apparent from studies with the GLP1R probes and GLP1R SNAP labels. Lastly, daLUXendin544 labelling was reduced ~50% in GLP1R^KO/KO^ islets, with a further reduction in labelling seen in the presence of a saturating concentration of GIPAib2 (1 µM) (Extended Data Fig. 2i,j).

### daLUXendins label most endocrine cell types within the islet

**Fig. 2:**
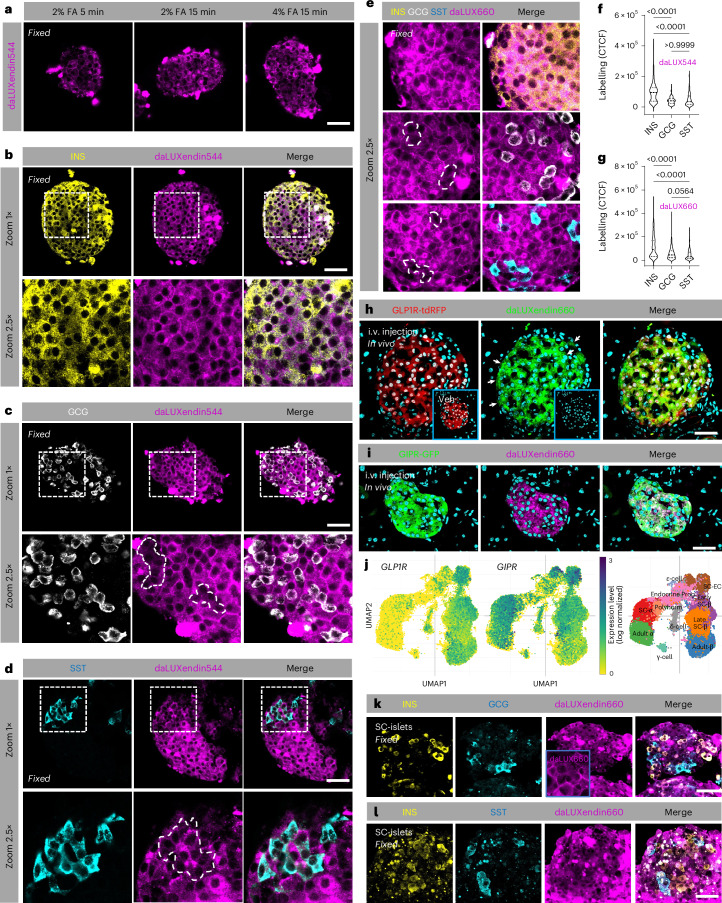
daLUXendin544 and daLUXendin660 reveal dual agonist targets in primary and SC-islets. **a**, Following labelling of live islets, daLUXendin660 can be fixed using either 2% or 4% formalin (FA) for 15 min (*n* = at least five islets from three mice). **b**, daLUXendin544 labels insulin-positive (INS^+^) cells throughout the mouse islet (*n* = 44 islets from five mice). **c**,**d**, daLUXendin544 labels glucagon-positive (GCG^+^) and somatostatin-positive (SST^+^) cells throughout the islet, although to a lesser extent than INS^+^ cells (INS, 44 islets from five mice; GCG, 25 islets from seven mice; SST, 29 islets from seven mice). **e**, daLUXendin660 displays a similar labelling pattern to daLUXendin544 in mouse islets (INS, 43 islets from five mice; GCG, 30 islets from seven mice; SST, 27 islets from seven mice). **f**,**g**, Quantification of daLUXendin544 (**f**) and daLUXendin660 (**g**) labelling intensity in INS^+^, GCG^+^ and SST^+^ cell populations (Kruskal–Wallis test with Dunn’s multiple comparisons test). CTCF, corrected total cell fluorescence. **h**, Intravenous (i.v.) injection of daLUXendin660, but not vehicle (inset), labels GLP1R^+^ cells in islets from GLP1R-tdRFP reporter mice (arrows show GLP1R^−^ but daLUXendin660+ cells) (*n* = at least nine islets). **i**, As in **h**, but with GIPR-GFP reporter mice (*n* = 9 islets). **j**, Uniform manifold approximation and projection (UMAP) plots showing *GLP1R* and *GIPR* expression in early and late SC-islet endocrine cell populations (taken from a previous publication^35^, and plotted using Single Cell Portal^54^, study SCP1526). **k**,**l**, daLUXendin660 labels INS^+^, GCG^+^ and SST^+^ cell populations in SC-islets (inset in **k**, a separate non-immunostained SC-islet showing membrane and cytoplasmic daLUXendin660 signal). Scale bar, 53 µm. Violin plots show min–max and median. Exact *P* values are displayed on each graph. Source data

To allow post hoc protein labelling and identification of cell types in complex tissue, we optimized a fixation protocol that retained strong and specific daLUXendin544 and daLUXendin660 labelling post-fixation with either 2% or 4% formalin for 15 min (Fig. 2a). Islets were labelled with daLUXendin544 before formalin-fixation, and immunostaining with antibodies against either insulin, glucagon or somatostatin to identify β cells, α cells or δ cells, respectively. As expected, given their abundant GLP1R and GIPR expression, β cells were strongly labelled with daLUXendin544 (Fig. 2b). Confirming results in live islets, α cells showed weaker but detectable daLUXendin544 labelling restricted to the cytoplasm (Fig. 2c), probably reflecting their abundant GIPR expression and largely absent GLP1R protein expression^17,18,20^. δ cells also showed weaker labelling versus β cells (Fig. 2d). However, labelling was observed both in the cytoplasm and membrane, which might reflect low-moderate expression of GIPR and GLP1R protein (Fig. 2d). Identical results were obtained with daLUXendin660 (Fig. 2e). Quantification of daLUXendin544/660 labelling is shown in Fig. 2f,g. Demonstrating efficacy of daLUXendin660 in vivo, islets in GLP1R-tdRFP and GIPR-GFP reporter mice were strongly and specifically labelled 60 min following intravenous injection with 100 nmol kg^−1^ probe but not vehicle (Fig. 2h,i). As for isolated islets, both GLP1R^+^ and GLP1R^−^ cells were labelled in vivo, the latter probably representing GIPR^+^ cells (that is, α cells) (Fig. 2h).

### daLUXendins label most cell types in human stem cell islets

Fluorescent labelling of low-abundance proteins in human islets is limited by high levels of autofluorescence, in particular from lipofuscin accumulation^32^. In addition, human islet preparations display heterogeneity in response to GLP1R/GIPR agonist, probably reflecting variable GLP1R/GIPR expression as well as time in culture. To circumvent these issues, we instead turned to human induced pluripotent stem (iPS) cell-derived islet-like structures (SC-islets), which are therapeutically important as a cell therapy for type 1 diabetes^33,34^. Analysis of published single-cell RNA sequencing data^35^ revealed *GIPR* and *GLP1R* in early and late β-like cells as well as δ cells, and *GIPR* in early and late α cells (Fig. 2j). daLUXendin660 labelling was qualitatively similar to that observed in mouse islets, with cells displaying two similar labelling patterns: membrane labelling + cytoplasmic labelling or cytoplasmic staining alone (Fig. 2k, inset). In keeping with the transcriptomic data, immunostaining revealed daLUXendin660 labelling in insulin (INS^+^), glucagon (GCG^+^) and somatostatin (SST^+^) cell populations (Fig. 2j–l). Some daLUXendin660 labelling was detected in INS^−^/GCG^−^/SST^−^ cells, which were presumably GLP1R/GIPR-expressing enteroendocrine cells that are present in most differentiations (Fig. 2j–l). Similar to mouse islets, the strongest daLUXendin660 labelling was seen in β cells, with weaker but detectable labelling in α cells and δ cells (Fig. 2k,l) (corrected total cell fluorescence, 3.60 × 10^5^ ± 2.58 × 10^5^ versus 2.30 × 10^5^ ± 1.59 × 10^5^ versus 2.16 × 10^5^ ± 1.46 × 10^5^ AU, β cells versus α cells versus δ cells, respectively; *P* < 0.0005 one-way ANOVA and Šídák’s multiple comparisons test) (*n* = 12 SC-islets from two differentiations).

### daLUXendin660+ optimized for long-term dosing

**Extended Data Fig. 3:**
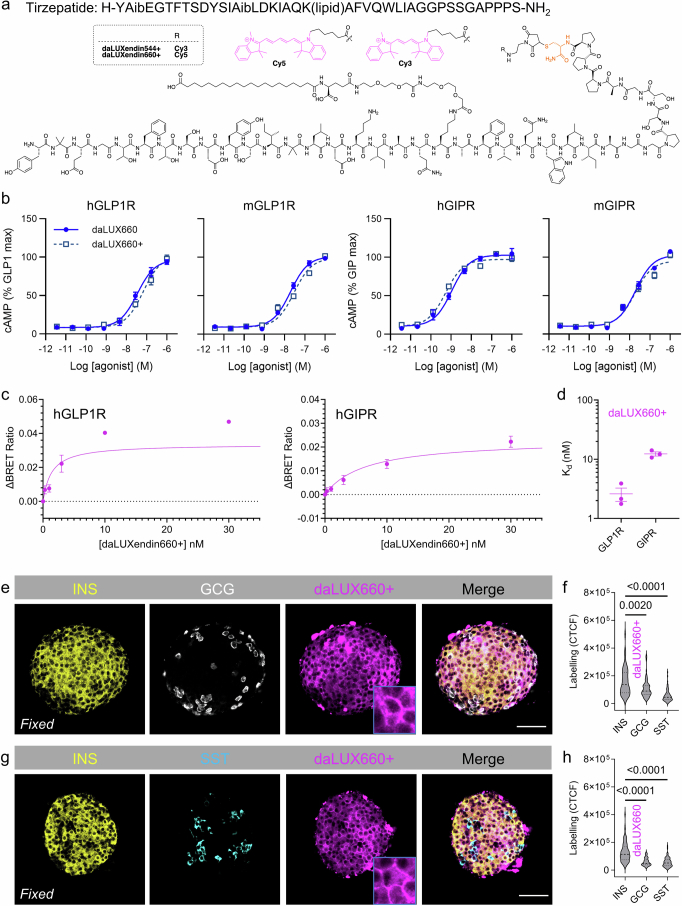
Development and characterization of lipidated daLUXendin660+. **a)** daLUXendin660 can be further modified to generate daLUXendin660+ by installing a C20 di-acid lipid chain at lysine position 20. **b)** cAMP responses to daLUXendin660 (daLUX660) and daLUXendin660+ (daLUX660+) in AD293 cells expressing either human (h)GLP1R, mouse (m)GLP1R, human (h)GIPR or mouse (m)GIPR (n = 4 independent repeats). **c, d)** Binding affinity of daLUXendin660+ against hGLP1R and hGIPR (**c**), with Kd values (**d**) in a separate bar graph (one-site fit specific binding) (n = 3 independent repeats). **e-h)** daLUX660+ labels islets similarly to daLUX660, with strength of labelling β cells > α cells = δ cells, as shown by representative images (**e**, **g**) and summary bar graphs (**f**, **h**) (INS, insulin = at least 26 islets from 5 mice; GCG, glucagon = at least 16 islets from 5 mice; SST, somatostatin = at least 16 islets from 5 mice) (Kruskal-Wallis test with Dunn’s multiple comparisons test). Scale bar = 53 µm. Scatter plots and line graphs show mean ± SEM. Violin plots shows min-max and median. Exact *p* values are displayed on each graph. Source data

To expand the utility of daLUXendin660 for long-term dosing, we installed a C20 di-acid lipid chain on lysine at position 20 to confer albumin binding. The new molecule, termed daLUXendin660+, performed similarly to daLUXendin660 in pharmacology and receptor binding assays, demonstrating that acylation and lipid modification is well tolerated (Extended Data Fig. 3a–d) (Extended Data Table 2 and Supplementary Table 4). No differences in pancreatic islet cell labelling could be detected between daLUXendin660 and daLUXendin660+ applied to the same islet batch (that is, INS > GCG and SST) (Extended Data Fig. 3e–h).

### daLUXendins label GLP1R^+^ and GIPR^+^ neurons

**Fig. 3:**
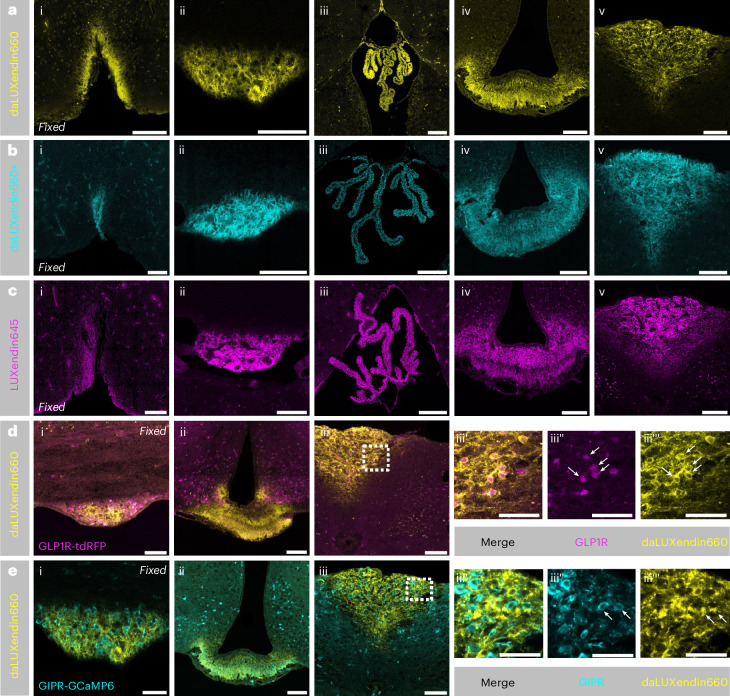
daLUXendin660 accesses circumventricular organs from the periphery. **a**–**c**, Intravenous administration of 100 nmol kg^−1^ daLUXendin660 (*n* = 3 mice) (**a**), 100 nmol kg^−1^ daLUXendin660+ (*n* = 2 mice) (**b**) and subcutaneous administration of 100 nmol kg^−1^ LUXendin645 (GLP1R antagonist) (*n* = 5 mice) (**c**) led to staining in circumventricular organs of the brain and the choroid plexus. Shown are the organum vasculosum of the lamina terminalis (i), subfornical organ (ii), choroid plexus (iii), median eminence (iv) and area postrema (v). **d**,**e**, Intravenous administration of 100 nmol kg^−1^ daLUXendin660 in a GLP1R-tdRFP mouse (*n* = 1) (**d**) or GIPR-GCaMP6 mouse (*n* = 2) (**e**) labels GLP1R-expressing and GIPR-expressing cells, respectively (indicated by white arrows). Shown are the subfornical organ (i), median eminence (ii), area postrema (iii) and zoom-in of area postrema with merged (iii’), GLP1R-tdRFP/GIPR-GCaMP6 (iii’’) and daLUXendin660 (iii’’’) signals. Note that images in **e** are from the same mouse as in **a** but with GIPR-GCaMP6 signal overlaid. Scale bars, 100 µm. Source data

Following intravenous injection (100 nmol kg^−1^), daLUXendin660 signal could be readily detected in the circumventricular organs and choroid plexus but not in other areas of the brain (Fig. 3a). The circumventricular organs are characterized by an incomplete blood–brain barrier and include areas such as the median eminence, area postrema, subfornical organ and organum vasculosum of the lamina terminalis, brain regions known to bind GLP1R and GIPR (ant)agonists^17,18^. We did not observe any difference in the extent of brain penetration between daLUXendin660, daLUXendin660+ and the single GLP1R agonist LUXendin645 (Fig. 3a–c). To establish which incretin receptor-expressing neurons are labelled with daLUXendin660, we performed intravenous injection (100 nmol kg^−1^) of daLUXendin660 in transgenic mice Cre-dependently expressing fluorescent reporters in either GLP1R-expressing cells (GLP1R-tdRFP) or GIPR-expressing cells (GIPR-GCaMP6). Clear colocalization of daLUXendin660 with GLP1R (Fig. 3d) and GIPR (Fig. 3e) neurons was observed in the area postrema.

**Extended Data Fig. 4:**
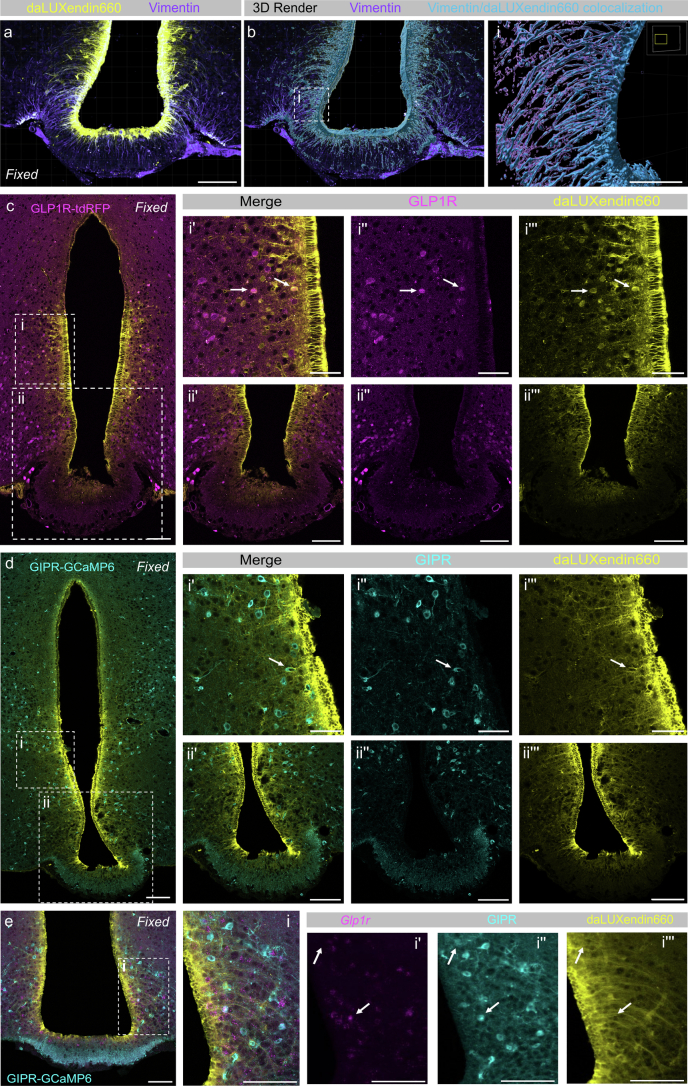
ICV-injected daLUXendin660 accesses the brain parenchyma and labels tanycytes. **a**) Intracerebroventricular (icv) injection of daLUXendin660 (3.3–5 nmol/kg BW) labels cells surrounding the 3rd ventricle. daLUXendin660 colocalizes with tanycytes (indicated by vimentin immunolabeling) following icv administration (n = 4 mice). **b**) 3D-rendering showing extensive colocalization of daLUXendin660 with vimentin in tanycytes surrounding the 3rd ventricle (box labelled with i indicates region of magnified image). Labelling of GLP1R- and GIPR-expressing cells (examples indicated by arrows) was characterized in **c**) GLP1R-tdRFP (n = 2) and **d**) GIPR-GCaMP6 (n = 2) mice. Boxes labelled with i and ii indicate region of magnified images, with ’, ’’ and ’’’ representing merged, GLP1R/GIPR and daLUXendin660 signals, respectively. **e**) A small number of cells positive for both GIPR and *Glp1r* are labelled with daLUXendin660 following icv administration (indicated by arrow heads). Box labelled with i indicates region of magnified image, with ’, ’’ and ’’’ representing *Glp1r*, GIPR and daLUXendin660 signals, respectively. Scale bar = 100 µm.

The strength and extent of labelling does not necessarily equate to agonist efficacy, especially for peptidic ligands that diffuse freely through fenestrated vessels of the median eminence into the ventricular system^22,36^. To assess the accessibility of daLUXendin660 binding sites from the cerebrospinal fluid, we performed intracerebroventricular injection of the probe. Intracerebroventricular administration of daLUXendin660 demonstrated clear penetration from the cerebrospinal fluid into the brain parenchyma. Strong labelling was observed in tanycytes—identified through vimentin immunostaining—within the third ventricle (Extended Data Fig. 4a). daLUXendin660 labelling in tanycytes was localized to the apical surface as well as cellular processes extending into the brain parenchyma (Extended Data Fig. 4b). Neurons identified as GLP1R^+^ in GLP1R-tdRFP reporter mice were co-labelled with daLUXendin660 in the vicinity of the ventricles (Extended Data Fig. 4c). Modest colocalization in GIPR^+^ neurons was evident in GIPR-GCaMP6 reporter mice (Extended Data Fig. 4d). A small number of neurons positive for both GIPR and GLP1R were labelled with daLUXendin660 (Extended Data Fig. 4e).

### daLUXendin660 engages highly ordered GLP1R/GIPR nanodomains

**Fig. 4:**
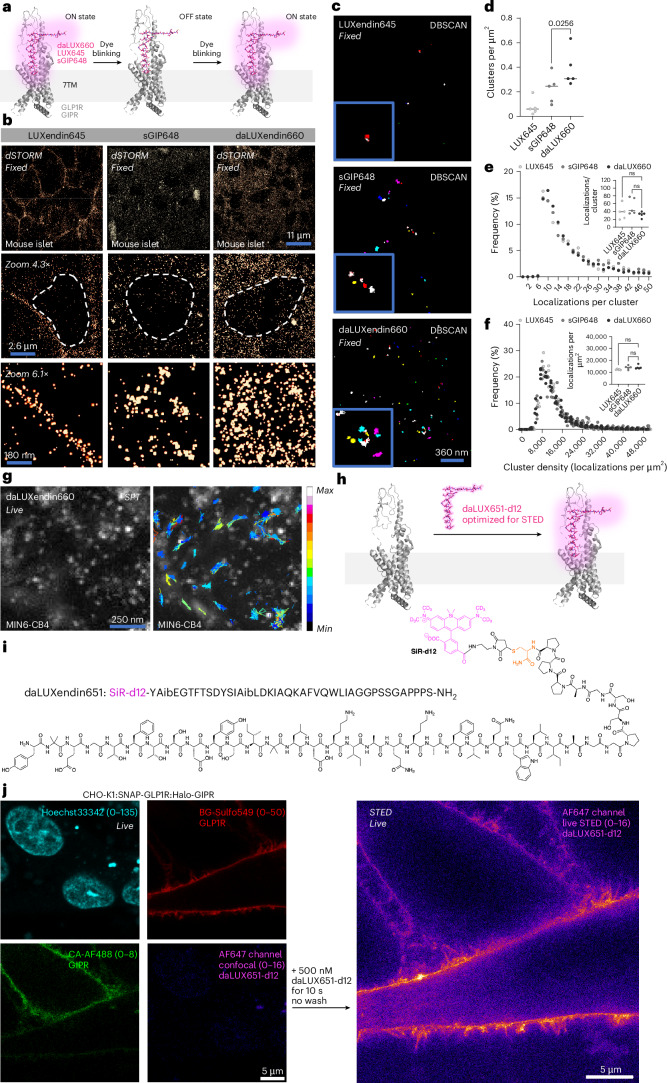
Dual agonist engages distinct endogenous GLP1R/GIPR nanodomains. **a**, Schematic showing single-molecule labelling and localization strategy for GLP1R (LUXendin645; LUX645), GIPR (sGIP648) and GLP1R/GIPR (daLUXendin660) (PDB 7VBI)^26^. **b**, Representative dSTORM images show different single-molecule densities and organization (30 nm) of LUX645-bound GLP1R, sGIP648-bound GIPR and daLUXendin660-bound GLP1R/GIPR across an islet cell population (nucleus is bounded by dashed line) (*n* = 5 islets from three mice). **c**,**d**, daLUXendin660 labels more GLP1R/GIPR clusters compared to either LUX645 or sGIP648, shown by representative images (**c**) and bar graph (**d**) (*n* = 5 islets from three mice) (one-way ANOVA with two-stage linear step-up procedure of Benjamini, Krieger and Yekutieli). **e**, Distribution and mean (inset) localization per cluster are similar across LUX645, sGIP648 and daLUXendin660 probes (*n* = 5 islets from three mice) (one-way ANOVA with Šídák’s multiple comparisons test). **f**, As in **e**, but for cluster density (one-way ANOVA with Šídák’s multiple comparisons test). **g**, Single particle tracking of daLUXendin660-labelled GLP1R/GIPR, showing min–max displacement (*n* = 5 cells, two independent repeats). **h**,**i**, SiR-d12 can be installed on daLUXendin (**h**) to create daLUXendin651-d12 (**i**), optimized for STED nanoscopy. **j**, Confocal snapshots showing CHO-K1:SNAP-GLP1R:Halo-GIPR cells labelled for GLP1R (BG-Sulfo549) and GIPR (CA-AF488) before live STED imaging of daLUXendin651-d12 labelling (*n* = 5 wells). Scale bars are provided on each figure. ns, non-significant. Exact *P* values are displayed on each graph. Source data

GPCRs possess higher organization at the cell membrane and within the cell, forming nanodomains that are important for signalling^16,37,38^. We therefore set out to determine how endogenous GLP1R/GIPR nanodomains might be targeted by single agonists versus a dual agonist in primary tissue; that is, pancreatic islets. We first established nanodomain organization in the non-stimulated but bound state using LUXendin645, a GLP1R antagonist coupled to Cy5. Following 60 min incubation with LUXendin645, fixation and dSTORM nanoscopy of whole islets, we confirmed our previous data showing that non-stimulated GLP1R form discrete nanodomains^16,18^ (Fig. 4a,b). The GIPR agonist sGIP648 also labelled discrete receptor nanodomains (GIPR), as defined by clustering (Fig. 4b–d). daLUXendin660, however, engaged more ordered receptor nanodomains compared to the GIPR agonist alone, with high levels of clustering probably reflecting increased receptor interactions (Fig. 4b–d). Localization number per cluster, as well as the density of each cluster, were similar across all ligands examined (Fig. 4e,f). Together, these data suggest that tirzepatide either engages GLP1R into clustering or increases interactions between GLP1R and GIPR, thus contributing to its unique nanodomain arrangement. Owing to the long acquisition times needed for localization counting, dSTORM is best suited to fixed tissue. We therefore set out to show the applicability of daLUXendin660 for TIRF-based GLP1R/GIPR single particle tracking at the cell membrane in live MIN6-CB4 cells (Fig. 4g). To open up super-resolution live imaging approaches, such as STED, we replaced the C-terminal Cy5 with fluorogenic deuterated silicon rhodamine (SiR-d12)^39^ to create daLUXendin651-d12 (Fig. 4h,i and Supplementary Fig. 1). Labelling with daLUXendin651-d12 was too dim to visualize endogenous receptor but was sufficient to map GLP1R in fixed CHO-K1:SNAP-GLP1R:Halo-GIPR cells. daLUXendin651-d12 was able to image GLP1R below the diffraction limit and could be recorded over multiple frames (Supplementary Fig. 2). Following direct application of daLUXendin651-d12 to CHO-K1:SNAP-GLP1R:Halo-GIPR cells, far-red cell surface signal from SiR-d12 became rapidly visible (10 s) in live STED on the cell surface, colocalizing with BG-Sulfo549 and CA-AF488 (Fig. 4j).

In the present study, we synthesize and validate daLUXendins, red and far-red GLP1R/GIPR dual agonist probes. Nanomolar concentrations of daLUXendin specifically label GLP1R/GIPR in live cells, allowing endogenous binding sites and cellular targets for dual agonists such as tirzepatide to be visualized and interrogated. daLUXendin labelling can be achieved in most cells and tissues with a simple 1 h incubation or injection, followed by washing and analysis. Advantageously, daLUXendins show minimal loss in signal intensity when formaldehyde-fixed, allowing co-labelling for other cellular or neuronal proteins and markers.

A key aspect of tirzepatide efficacy is increased potency and selectivity at human GIPR compared to GLP1R, with approximately 70% of activity at GIPR^10–12^. In mice, however, tirzepatide is selective for GLP1R over GIPR, and its insulinotropic and overall therapeutic efficacy is equivalent or non-superior to semaglutide (GLP1R agonist)^10,11^. Although this is unlikely to have any bearing on GLP1R and GIPR accessed and bound by tirzepatide, it remains a limitation to the use of preclinical mouse models to understand GLP1R/GIPR dual agonism. Notably, daLUXendin544 and daLUXendin660 displayed less selectivity for GLP1R over GIPR in mouse (1:2 GIPR:GLP1R) compared to tirzepatide (1:8 GIPR:GLP1R). Although the exact mechanisms underlying the differences between daLUXendin544/660 and tirzepatide remain unknown, our previous studies showed that fluorophore modification can increase GLP1R agonist efficacy^18^. Therefore, daLUXendin660 could be a useful tool for probing dual agonist biology in the mouse.

Within the pancreatic islet, tirzepatide labelled β cells > α cells = δ cells, in keeping with known protein or transcript expression levels for GLP1R and GIPR (as previously reviewed^21^). Although studies have shown that tirzepatide stimulates secretion of insulin, somatostatin and glucagon^11,21^, the cellular substrates have so far remained elusive. The observation that tirzepatide labels β cells, δ cells and α cells suggests both direct and paracrine modes of action. Given that α cells express GIPR >> GLP1R, direct effects of tirzepatide on glucagon secretion are probably mediated by GIPR signalling, a known stimulator of α cell function^40,41^. By contrast, δ cells express GIPR > GLP1R, inferring a predominant role for GIPR in tirzepatide-stimulated somatostatin secretion, with some GLP1R contribution. β cells express GLP1R = GIPR, but increased affinity of tirzepatide for human GIPR^10^ is likely to disproportionately contribute to insulin secretion. As well as exerting direct effects on islet cell function, tirzepatide is also likely to stimulate α cell-to-β cell and δ cell-to-β cell communication, further regulating insulin release (as previously reviewed^21,42^). At the same time, tirzepatide might engage β cell-to-α cell or δ cell-to-α cell communication to provide a brake on glucagon secretion.

Peripherally injected daLUXendin660 was able to label the pancreas as well as readily access the median eminence, organum vasculosum of the lamina terminalis, subfornical organ and area postrema. The extent of daLUXendin660 labelling was similar to that seen for a GLP1R antagonist, LUXendin645, as well as a GIPR agonist, sGIP648 (refs. ^17,18^). We therefore speculate that the superior efficacy of GLP1R/GIPR dual agonism versus single GLP1R agonism reflects the specific neuronal and supporting cell populations that are activated rather than the extent of brain access. When administered directly into the ventricular space of the brain, daLUXendin660 labelled GLP1R^+^ and GIPR^+^ neurons in the vicinity of the ventricles, as well as periventricular cells such as third ventricular tanycytes. Periventricular cell labelling is a common feature across GLP1R/GIPR (dual) (ant)agonists, pointing to a critical role for this cell type in the regulation of food intake and possibly the ligands themselves^43^.

Outside of conventional pharmacology, it remains unknown how daLUXendin660 interacts with GLP1R/GIPR in cells that endogenously express both receptors. Single-molecule localization microscopy showed that daLUXendin660 densely labelled GLP1R/GIPR clusters or nanodomains in islets, which was in addition to that seen with a GIPR agonist alone. Therefore, daLUXendin660 probably influences either GLP1R clustering or GLP1R and GIPR localization to increase nanodomain formation. We speculate that GIPR and GLP1R signalling within or between cell nanodomains, rather than simple signal summation across the cytoplasm, contributes to tirzepatide efficacy, as recently shown for GLP1 and β_2_-adrenergic receptors^44^.

Tirzepatide is just one of many emerging dual and triple agonists that might exhibit different receptor engagement profiles. Given that we are still piecing together single agonist function^45^, daLUXendin provides a powerful tool to understand dual agonist receptor engagement, brain or organ access and receptor synergism. Owing to the structural similarities between dual and triple agonists, in the future, daLUXendins can be easily modified to incorporate a glucagon receptor agonist component.

The study has a number of limitations. Firstly, tirzepatide and daLUXendins are different molecules, and detailed pharmacokinetic profiles were not determined. Therefore, we cannot categorically state that brain areas or cell types accessed by tirzepatide in humans and rodents would be similar to those accessed by daLUXendins in rodents. Secondly, we did not provide any data on human islets. We have noticed heterogeneity in GLP1R/GIPR expression, probably reflecting culture time and donor variability, compounded by high levels of lipofuscin autofluorescence^32^. Thirdly, we restricted daLUXendin544/660/651-d12 to Cy3, Cy5 and SiR-d12 fluorophores, as they are well suited for complex tissue labelling^16,18,19^. In the future, the daLUXendin colour palette could be extended to include blue-near-infrared and even epitope tags to allow signal amplification.

In summary, daLUXendin544/660 are highly specific probes that highlight dual agonist target cells within complex tissues such as the pancreatic islet and brain. We envisage that daLUXendin544/660 will provide interrogable mechanisms underlying dual agonist efficacy.

## Methods

### Study design

Individual data points are reported in the figures, and no data were excluded. Islet isolation is a nuisance variable, and as such, data are taken from independent islet preparations. To ensure that all states were represented in the different experiment arms, randomization was used to allocate samples and animals to treatment groups. All studies combined males and females, as no sex-specific phenotype has been reported for any of the genetically modified lines used, and male and female tissues were indistinguishable by their chemical probe or SNAP-tag labelling.

### Ethics

Animal studies were regulated by the Animals (Scientific Procedures) Act 1986 of the UK (Personal Project Licences P2ABC3A83, PP1778740 and PP6526002). Approval was granted by the University of Birmingham, University of Oxford and University College London Animal Welfare and Ethical Review Bodies (AWERB).

Studies with human iPS cells were reviewed by the University of Oxford Medical Sciences Interdivisional Research Ethics Committee (MS IDREC). The study utilizes iPS cells, which are created entirely outside the body and, as such, do not fall under the Governance of the Human Tissue Authority requiring ethics approval for their use in the United Kingdom.

### General chemistry

Chemicals and solvents were purchased from Merck, TCI (Tokyo Chemical Industry) and Acros Organics (Thermo Fisher Scientific) and used without further purification. Dry solvents were purchased from Acros Organics (Thermo Fisher Scientific). Amino acids and resins for solid-phase peptide synthesis were purchased from Novabiochem (Merck) or Iris Biotech.

Ultra-high performance liquid chromatography with UV–visible light detection was performed for purity assessment, using an Agilent 1260 Infinity II LC System equipped with an Agilent SB-C18 column (1.8 µm, 2.1 × 50 mm). Buffer A consisted of 0.1% formic acid in H_2_O; buffer B consisted of 0.1% formic acid in acetonitrile. The typical gradient was from 10% B for 1.0 min → gradient to 95% B over 5 min → 95% B for 1.0 min with 0.45 ml min^−1^ flow or from 30% B for 1.0 min → gradient to 95% B over 5 min. Retention times are given in minutes. Chromatograms were imported into Graphpad Prism 8, and purity was determined by calculating area under the curve ratios.

Preparative or semi-preparative high-performance liquid chromatography was performed on an Agilent 1260 Infinity II LC System equipped with columns as follows: preparative column, Reprospher 100 C18 columns (10 μm: 50 × 30 mm at 20 ml min^−1^ flow rate; semi-preparative column, 5 μm: 250 × 10 mm at 4 ml min^−1^ flow rate. Eluents A (0.1% TFA in H2O) and B (0.1% TFA in MeCN) were applied as a linear gradient. Peak detection was performed at maximal absorbance wavelength.

For high-resolution mass spectrometry, samples were analysed on an Orbitrap Fusion mass spectrometer (Thermo Fisher Scientific). Mass spectrometry scans were acquired in a range of 350–1500 *m*/*z*. MS1 scans were acquired in the Orbitrap with a mass resolution of 120,000 with an AGC target value of 4 × 10^5^ and 50 ms injection time. MS2 scans were acquired in the ion trap with an AGC target value of 1 × 10^4^ and 35 ms injection time. Precursor ions with charge states two to four were isolated with an isolation window of 1.6 *m*/*z* and 40 s dynamic exclusion. Precursor ions were fragmented using higher-energy collisional dissociation with 30% normalized collision energy.

### Peptide synthesis

Peptide synthesis and chemical characterization (high-resolution mass spectrometry, high-performance liquid chromatography) are detailed in the Supplementary Note 1.

### HTRF cAMP assay

AD293 cells (RRID:CVCL_9804) were transiently transfected 24 h before the assay in six-well plates with plasmids encoding wild-type hGLP1R, mGLP1R, hGIPR or mGIPR using Lipofectamine 2000. AD293 cells were authenticated at source using STR profiling. Cells were detached and treated with a range of agonist concentrations in serum-free DMEM + 0.1% BSA for 30 min at 37 °C in 96-well plates, followed by the addition of lysis buffer 2 (Cisbio) at a 1:2 ratio. Lysates were further diluted 1:10 to avoid spectral interference from the higher concentrations of fluorescent ligands in the HTRF assay, and the concentration of cAMP was determined after the addition of cAMP Dynamic detection reagents (Cisbio). Three-parameter logistic fitting was used to determine signalling potencies for each ligand, with EC_50_ ratios relative to the cognate ligand for each receptor used to establish test agonist selectivity for GLP1R versus GIPR.

### NanoBRET binding assays

HEK293T cells (RRID:CVCL_0063) were cultured in DMEM/F12 GlutaMAX (ThermoFisher), supplemented with 10% FBS and 1% antibiotic-antimycotic solution and cultured at 37 °C, 5% CO_2_. HEK293T cells were authenticated at source using STR profiling. Cells transiently expressing Nluc–GLP1R or Nluc–GIPR were seeded onto white 96-well plates and cultured for 24 h. Media was then removed and the cells were incubated in PBS supplemented with 0.1% BSA and 0.01% NanoGlo (Promega). For saturation binding assays, daLUXendins were added across the concentration range 0.1–31.6 nM, in the absence or presence of 1 μM GIP and 1 μM Exendin-9 to determine specific binding. For competition binding assays, cells were co-treated with 10 nM daLUXendin and GLP1 or GIP across the range 1 μM to 1 pM. Plates were read using a PheraSTAR microplate reader, using the NanoBRET filter module. BRET ratios (λ_acceptor_/λ_donor_) were calculated, and saturation data fit using the ‘One Site – Specific Binding’ equation of GraphPad Prism 10. Competition binding was fit using the ‘One Site – Fit Ki’ equation.

### Glucose tolerance testing

Male and female 8–12 week old *Glp1r*^WT/WT^, *Glp1r*^WT/KO^ and *Glp1r*^KO/KO^ (ref. ^18^) littermates (on a C57BL/6J background) were fasted for 4–6 h, with free access to water. Wild-type and heterozygous mice were used as controls, as loss of a single *Glp1r* allele was found to exert minimal influence on phenotype^46^. Tirzepatide and daLUXendin were administered at 10 nmol kg^−1^ before intraperitoneal injection of 2 g kg^−1^ glucose 60 min later. Glucose was measured in blood from the tail vein at 0, 15, 30, 60, 90 and 120 min post glucose challenge. Mice were socially housed in specific-pathogen-free conditions under a 12 h light–dark cycle with ad libitum access to food and water, relative humidity 55 ± 10% and temperature 21 ± 2 °C.

### Food intake study

Male and female 12–14 week old C57BL/N mice were singly housed in a 12 h dark–light cycle (14:00–02:00 h) and habituated to FED3 feeding devices for 7 days before commencing the study^47^. All mice were kept on a 12 h light–dark cycle at 20–24 °C and 45–65% relative humidity (typically 21 °C and 55%). On test days, mice were fasted for 3 h before onset of the dark phase by removal of the FED3 devices. At dark onset, mice were injected subcutaneously with either vehicle, daLUXendin660 (10 nmol kg^−1^) or tirzepatide (10 nmol kg^−1^), the FED3 devices were returned and food intake was recorded over a 24 h period. The study was performed using a three-way crossover design. A 1-week washout period was observed between experimental days. FED3 devices were checked, serviced and food was topped up daily.

### AD293 cell fluorescent probe labelling

AD293 cells (RRID:CVCL_9804) were grown in DMEM High Glucose supplemented with 10% FBS, 100 units per ml penicillin and 100 μg ml^−1^ streptomycin and 1% glutamine (complete DMEM).

Cells were transfected with 0.3 μl Lipofectamine 2000 per well and 50 ng DNA (SNAP_hGIPR or SNAP_hGLP1R plasmid) per well in 110 μl OptiMEM per well and incubated overnight at 37 °C, 5% CO_2_. Cells co-transfected with plasmids for two receptors harbouring orthogonal enzyme self-labels (SNAP_GIPR and Halo_GLP1R plasmid) received 50 ng of each DNA. Control, non-transfected cells were cultured overnight in 50 μl OptiMEM without the addition of Lipofectamine 2000 or DNA. The next day, culture media was removed and 50 μl of labelling solution or complete DMEM (for unlabelled control) was added to each well.

On the day after transfection with SNAP-GIPR and Halo-GLP1R, cells were incubated in triple probe solutions of either 500 nM daLUXendin544 with 500 nM SBG-Oregon Green (SBG-OG) and 500 nM CA-Sulfo646, or 500 nM daLUXendin660 with 500nM SBG-OG and 500 nM CA-Sulfo549, all in 50 μl complete DMEM for 55 min at 37 °C, 5% CO_2_. Additional wells of transfected cells were incubated in single probe solutions (500 nM daLUXendin544, 500 nM daLUXendin660, 500 nM SBG-OG, 500 nM CA-Sulfo549 or 500 nM CA-Sulfo646) or unlabelled complete DMEM in addition to non-transfected cells incubated in triple probe solutions, all as control wells. SNAP-tag and Halo-tag have been previously reported and are characterized elsewhere^30^.

### MIN6-CB4 cell fluorescent probe labelling

MIN6-CB4 cells were generated and phenotyped, as previously described^48^. Cells were grown to 70% confluency in MIN6 media (DMEM High Glucose supplemented with 15% FBS, 71 μM 2-mercaptoethanol, 2 mM glutamine, 100 units ml^−1^ penicillin, 100 μg ml^−1^ streptomycin). Cells were then seeded in 100 μl MIN6 media per well, on a glass-bottomed 96-well imaging plate previously coated with poly-l-lysine 0.01%. The day after seeding, cells were incubated with either 500 nM daLUXendin660, 100 nM sGIP549 (GIPR probe)^17^ or 100 nM LUXendin551 (GLP1R probe)^18^, a combination of two or just complete MIN6 media for 1 h at 37 °C, 5% CO_2_.

### Islet isolation

Male and female 8–12 week old CD1 and C57BL6/J mice, as well as GLP1R^KO/KO^ (ref. ^18^) and GLP1R^SNAP/SNAP^ (ref. ^16^) mice (both on a C57BL/6J background) were used as tissue donors. Mice were humanely killed using a schedule-1 method, and the common bile duct was injected with Serva NB8 (1 mg ml^−1^) collagenase. The inflated pancreas was dissected, and islets were isolated using Histopaque-1119 and 1083 (Sigma-Aldrich) gradients and cultured at 37 °C, 5% CO_2_ in RPMI supplemented with 10% FBS (Gibco), 100 units per ml penicillin and 100 μg ml^−1^ streptomycin. Islets were used up to 4 days after isolation.

### Islet labelling and immunostaining

Size-matched wild-type or GLP1R^KO/KO^ islets were incubated in 100 nM, 500 nM or 1 μM daLUXendins, sGIP549, sGIP648, LUXendin551 or LUXendin645 for 1 h at 37 °C, 5% CO_2_, before washing twice.

GLP1R^SNAP/SNAP^ islets were incubated with 500 nM BG-Sulfo549 or BG-Sulfo646 and/or another orthogonal fluorescent ligand for 1 h at 37 °C, 5% CO_2_, before washing three times.

For immunostaining, labelled islets were washed three times with PBS, then incubated with 2–4% formalin for 15 min at room temperature (16–25 °C). Islets were again washed three times with PBS, then incubated in permeabilizing blocking buffer for 1 h at room temperature before incubation with primary antibody overnight at 4 °C. The following day, islets were incubated with secondary antibody for 2 h at room temperature before mounting onto microscope slides with VECTASHIELD HardSet with DAPI or Everbrite Hardset. Primary and secondary antibodies are listed in Supplementary Table 5.

### Brain and pancreas labelling

To assess brain and pancreas access following peripheral administration, GLP1R-Cre:tdRFP (GLP1R-tdRFP) and GIPR-Cre:GFP (GIPR-GFP) mice^15^ (both on a C57BL/N background) were injected intravenously into the tail vein with 100 nmol kg^−1^ daLUXendin. Then, 40 min later, mice were terminally anaesthetized with pentobarbital (200 mg kg^−1^) and perfused with ice-cold 0.1 M PBS followed by 4% formalin solution.

For brain tissue analysis, GLP1R-tdRFP mice and GIPR-Cre:GCaMP6 (GIPR-GCaMP6) mice^14^ were terminally anaesthetized using ketamine hydrochloride (Ketavet, Zoetis; 75 mg kg^−1^, intraperitoneally) and medetomidine hydrochloride (Domitor, OrionPharma; 1 mg kg^−1^, intraperitoneally). Meloxicam (Metacam, Boehringer Ingelheim; 5 mg kg^−1^, subcutaneously) was given for peri-surgery analgesia. Mice were placed in a stereotaxic frame, and daLUXendin was injected unilaterally into the lateral ventricle at doses of 5 nmol kg^−1^ body weight at a rate of 1 µl min^−1^. Coordinates relative to bregma were A/P −0.5 mm; D/V −2.5 mm; M/L 1 mm. At 1 h post injection, mice were perfused with ice-cold 0.1 M PBS followed by 4% formalin solution.

All brains were cryo-sectioned coronally at 30 µm and mounted onto microscope slides with VECTASHIELD antifade mounting medium (H-1000).

### Immunofluorescence labelling in brain tissue

Brains were cryo-sectioned coronally at 30 µm and sections processed for amplification of fluorescent reporter signals by immunofluorescence labelling of vimentin (a marker for ependymal cells and tanycytes). Antigen retrieval of free-floating sections was performed using sodium citrate buffer at 80 °C for 20 min. Sections were next blocked in 5% normal donkey serum, 0.2% Triton X-100 for 1 h and then incubated overnight in primary antibody (in blocking solution) at room temperature (primary antibody: chicken anti-vimentin, Ab24525, Abcam; 1:750). Sections were next incubated for 2 h in secondary antibody (in 1% normal donkey serum, 0.2% Triton X-100) at room temperature (secondary antibody: donkey anti-chicken IgY (H+L) highly cross-adsorbed secondary antibody, Alexa Fluor 488, A78948, Thermo Fisher Scientific; 1:500). Sections were stained with DAPI to mark nuclei, then mounted on Superfrost Plus slides and cover-slipped using Prolong Antifade medium.

Sections were imaged on an inverted Leica SP8 confocal microscope using either a ×25 or ×63 oil immersion objective. Three-dimensional reconstructions of daLUXendin660-labelled tissue or antibody-stained cells were rendered using the Surfaces function in Imaris (v.10.1.1) (Oxford Instruments). Opaque surfaces are three-dimensional reconstructions of overlapping areas of daLUXendin660 labelling and antibody staining created using the Coloc function in Imaris. Primary and secondary antibodies are listed in Supplementary Table 5.

### RNAscope in situ hybridization

Midbrain sections (30 µm) containing the ARH from a GIPR-Cre:GCaMP6 (GIPR-GCaMP6) mouse intracerebroventricularly administered daLUXendin660 (3.3 nmol kg^−1^ body weight) were collected on Superfrost Plus slides and allowed to air-dry at room temperature for 1 h. Slides were then dipped in molecular-grade ethanol and further air-dried overnight at room temperature. RNAscope in situ hybridization was performed on these sections using the RNAscope Multiplex Fluorescent Kit (v.2) (Advanced Cell Diagnostics) as per the manufacturer’s instructions, with a modification to the pre-treatment procedure (Protease IV incubation conducted for 20 min at room temperature). Sections were processed for in situ hybridization of *Glp1r* mRNA (cat. no. 418851-C2, Advanced Cell Diagnostics). Following hybridization, slides were cover-slipped using Prolong Antifade medium.

### iPS cell-derived islet-like structures

Human iPS cells (ALSTEM, iPS11) were maintained on iPS cell-qualified Matrigel (Corning, 354277) in mTeSR+ media (StemCell Technologies, 05826). ALSTEM iPS11 cells were authenticated at source by testing for expression of OCT4 and TRA-1-60, as well as alkaline phosphatase activity. Differentiation to islet-like structures (SC-islets) was carried out in a suspension-based, magnetic CELLSPIN bioreactor system (PFEIFFER). Differentiation media was changed daily by letting spheres settle by gravity for 3–10 min. Most of the supernatant was removed by aspiration; fresh media was added and bioreactors were placed back on the stirrer system. SC-islet differentiation was based on published protocols^35,49^

### Microscopy

Live or fixed cell and tissue imaging was performed with either (1) Zeiss LSM780/LSM880 meta-confocal microscopes equipped with GaAsP spectral detectors and ×40 and ×63/1.2 NA water objectives; (2) an Olympus FV3000 confocal microscope equipped with GaAsP spectral detectors and UPLSAPO ×60/1.30 NA silicone and ×60/1.41 NA oil objectives; or (3) an Olympus FV4000 confocal microscope equipped with SilVIR spectral detectors and a UPLSAPO ×60/1.41 NA oil objective. Excitation (ex) and emission (em) wavelengths (in nm) are as follows: sGIP549 (λex 561, λem 568–621), sGIP648 (λex 633, λem 639–692), LUXendin551 (λex 561, λem 568–621), LUXendin645 (λex 633, λem 639–692), daLUXendin544(+) (λex 561, λem 569–623), daLUXendin660(+) (λex 633, λem 640–694), SBG-OG (λex 488, λem 496–542), BG-Sulfo549 (λex 561, λem 569–623), BG-Sulfo646 (λex 633, λem 641–694), CA-Sulfo549 (λex 561, λem 568–613), CA-Sulfo646 (λex 633, λem 640–694), Hoechst (λex 405, λem 410–488), Alexa488 (λex 488, λem 496–542), DyLight488 (λex 488, λem 496–542) and DyLight633 (λex 633, λem 640–694). Zen 2012 or Olympus cellSens (v.3.2) were used for image acquisition and analysis.

### dSTORM nanoscopy

Islets were labelled with LUXendin645 (500 nM), sGIP648 (1 µM) or daLUXendin (1 µM), before fixation in 2–4% formalin for 15–30 min. Islets were mounted on cavity slides, submerged in STORM buffer (Abbelight) and sealed using a 170 µm coverslip and dental resin. Samples were imaged in HILO mode on an Evident/Abbelight SAFe 180 system, using a ×100/1.5 NA Olympus UPLAPO100XOHR objective. LUXendin645, sGIP648 or daLUXendin660 (all Cy5) were pumped to the dark state using an Oxxius laser combiner and 600 mW 640 nm Coherent Obis diode laser before initiation of photoblinking. Single-molecule events were recorded using an LP650 filter with an integration time of 50 ms on a Hamamatsu ORCA-Fusion sCMOS for 20,000–40,000 frames. Localizations were extracted and images reconstructed using Abbelight NEO software. Density-based spatial clustering of applications with noise (DBSCAN) was used to determine localization clustering, implemented in Abbelight NEO software (v.39), with ε = 25 nm (the average precision of the data) and minPts = 8. Results were confirmed using a custom RNA segmentation and DBSCAN routine implemented in R Project (v.4.4.3) (https://cran.r-project.org/web/packages/dbscan/index.html)^50^.

### Live confocal and STED imaging

Live confocal and STED imaging of CHO-K1:SNAP-GLP1R:Halo-GIPR cells (CHO-K1, RRID:CVCL_0214) was performed on a STEDYCON system (Abberior Instruments), mounted on a Nikon Eclipse TI research microscope equipped with a Plan APO Lambda ×100/1.45 NA oil objective (Nikon) and controlled by NIS Elements (Nikon). CHO-K1 cells were authenticated at source using DNA barcoding and DNA profiling. An incubation chamber surrounding the microscope setup was set to 37 °C 24 h before live-cell imaging. To provide stable focus during imaging, the Perfect Focus System (Nikon) was used. Imaging was performed in FluoroBrite Medium (Gibco) at 37 °C. Excitation was delivered with a 405, 488, 568 or 640 nm diode laser, and emission was detected with avalanche photodiodes at 461, 520, 566 or 671 nm, respectively. Live STED images were acquired with a 640 nm excitation laser, 775 nm depletion laser (at 20% intensity) and 671 nm detector (gate, 1–7 ns). Pixel size was set to 30 nm × 30 nm, with 10 µs pixel dwell time, and a line accumulation of 1 was used during acquisition. STEDYCON 9.0.799-g22f03ed2 software was used for image acquisition and analysis.

### Single particle tracking

MIN6-CB4 cells were labelled with 500 pM daLUXendin660 in complete media for 20 min at 37 °C and washed three times in HEPES-bicarbonate buffer, ensuring sparse labelling of GLP1R/GIPR. Cells were mounted on cavity slides and imaged in HEPES-bicarbonate buffer supplemented with 11 mM d-glucose before TIRF imaging at 40 Hz using an Evident/Abbelight SAFe 180 system and a ×100/1.5 NA Olympus UPLAPO100XOHR objective. Single particles were recorded using an LP650 filter with an integration time of 25 ms on a Hamamatsu ORCA-Fusion sCMOS for 1,750 frames. Single particle analysis was performed using the Trackmate plugin for ImageJ^51^, with trajectories shown as maximum displacement.

### Image analysis

Probe labelling was analysed using corrected total cell fluorescence, which is integrated density − (area of selected cell × mean fluorescence of background readings)^52,53^. Colocalization was determined using Manders’ coefficient, which calculates the proportion of pixels in one channel that also show intensity from both channels. Full width at half maximum was used to analyse labelling patterns at the membrane versus cytoplasm. All analyses were performed in ImageJ (NIH), with brightness and contrast linearly adjusted across the entire image for presentation purposes and applied equally between all states under examination.

### Statistics and reproducibility

Data distribution was assessed using the D’Agostino–Pearson normality test. Pairwise comparisons were made using unpaired *t*-tests. For non-parametric data, pairwise comparisons were made using a Mann–Whitney test. Multiple comparisons were made using either one-way ANOVA (parametric), two-way ANOVA (parametric) or Kruskal–Wallis test (non-parametric), with pairwise comparisons made using either Šídák’s or Dunn’s tests, or the two-stage linear step-up procedure of Benjamini, Krieger and Yekutieli. GraphPad Prism 9 and 10 were used for all statistical analyses. Values are presented as means; error bars, s.e.m.; *P* values less than 0.05 were considered significant.

For animal studies, each independent replicate corresponds to a separate animal. For cell line studies, the independent replicate corresponds to experiments performed on different days or using cells split from separate flasks. Variation between individual islets or cells was greater than variation of the mean between animals; therefore, they are considered separate data points for analysis purposes. For representative images, the experiment was repeated the same number of times as the related quantification, always with similar results. If the *n* number occupies a range of samples or animals, the lowest value is provided as per journal guidelines. Hence, the number of data points on the graph may be higher than the stated sample size.

### Reporting summary

Further information on research design is available in the Nature Portfolio Reporting Summary linked to this article.

## Supplementary information

Supplementary Information Supplementary Figs. 1 and 2, Supplementary Tables 1–5, Supplementary Note 1

Reporting Summary

Supplementary Data 1 Statistical Source Data for Supplementary Fig. 2

## Source data

Source Data Fig. 1 Statistical Source Data

Source Data Fig. 2 Statistical Source Data

Source Data Fig. 4 Statistical Source Data

Source Data Extended Data Fig./Table 1 Statistical Source Data

Source Data Extended Data Fig./Table 2 Statistical Source Data

Source Data Extended Data Fig./Table 3 Statistical Source Data

## Figures and Tables

**Extended Data Table 1:**
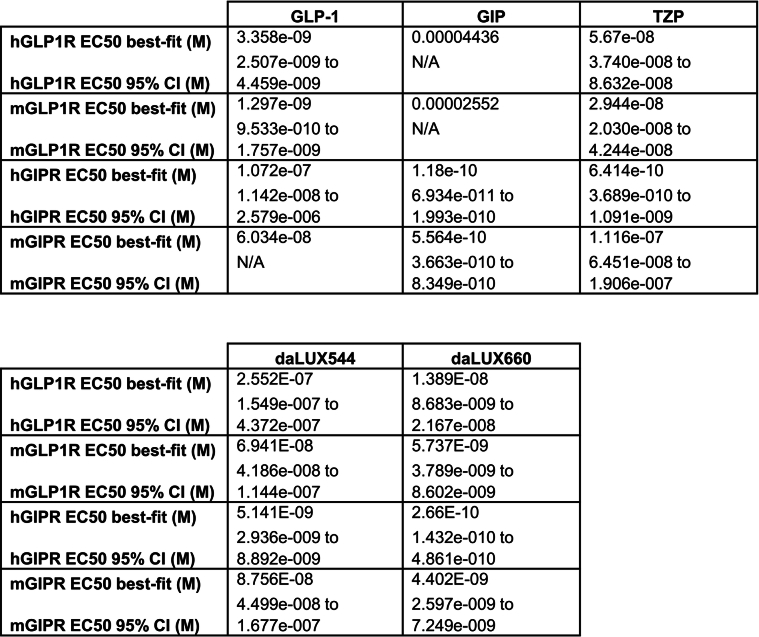
daLUXendin554 and daLUXendin660 cAMP pharmacology

*EC*_50_ values for GLP1, GIP, Tirzepatide (TZP), daLUX544 and daLUX660 against human GLP1R (hGLP1R), mouse GLP1R (mGLP1R), human GIPR (hGIPR) and mouse GIPR (mGIPR) (n = 4 independent repeats).

**Extended Data Table 2:**
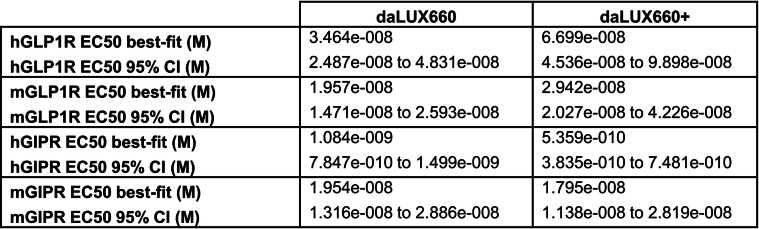
daLUXendin660 and daLUXendin660+ pharmacology

cAMP *EC*_50_ values for daLUX660 and daLUX660+ against human GLP1R (hGLP1R), mouse GLP1R (mGLP1R), human GIPR (hGIPR) and mouse GIPR (mGIPR) (n = 4 independent repeats).

## Data Availability

Datasets generated and/or analysed during the current study are available from the corresponding authors upon request. Owing to their large size, individual raw image files are available upon request. daLUXendins are subject to a Material Transfer Agreement and the ability to manufacture and supply. All requests for reagents and data will be handled by J.B. or D.J.H., who will respond within 30 working days. Source data are provided with this paper.
