# Supplementary material for: Fluorescent GLP1R/GIPR dual agonist probes reveal cell targets in the pancreas and brain
Source: Nat Metab. 2025 Aug 19;7(8):1536–49. doi: 10.1038/s42255-025-01342-6 (PMC12373499; doi:10.1038/s42255-025-01342-6)

nature metabolism

Supplementary information https://doi.org/10.1038/s42255-025-01342-6

Fluorescent GLP1R/GIPR dual agonist probes reveal cell targets in the pancreas and brain

In the format provided by the authors and unedited

Table of contents

Supplementary Figure 1

Supplementary Figure 2

Supplementary Table 1

Supplementary Table 2

Supplementary Table 3

Supplementary Table 4

Supplementary Table 5

Supplementary Note 1

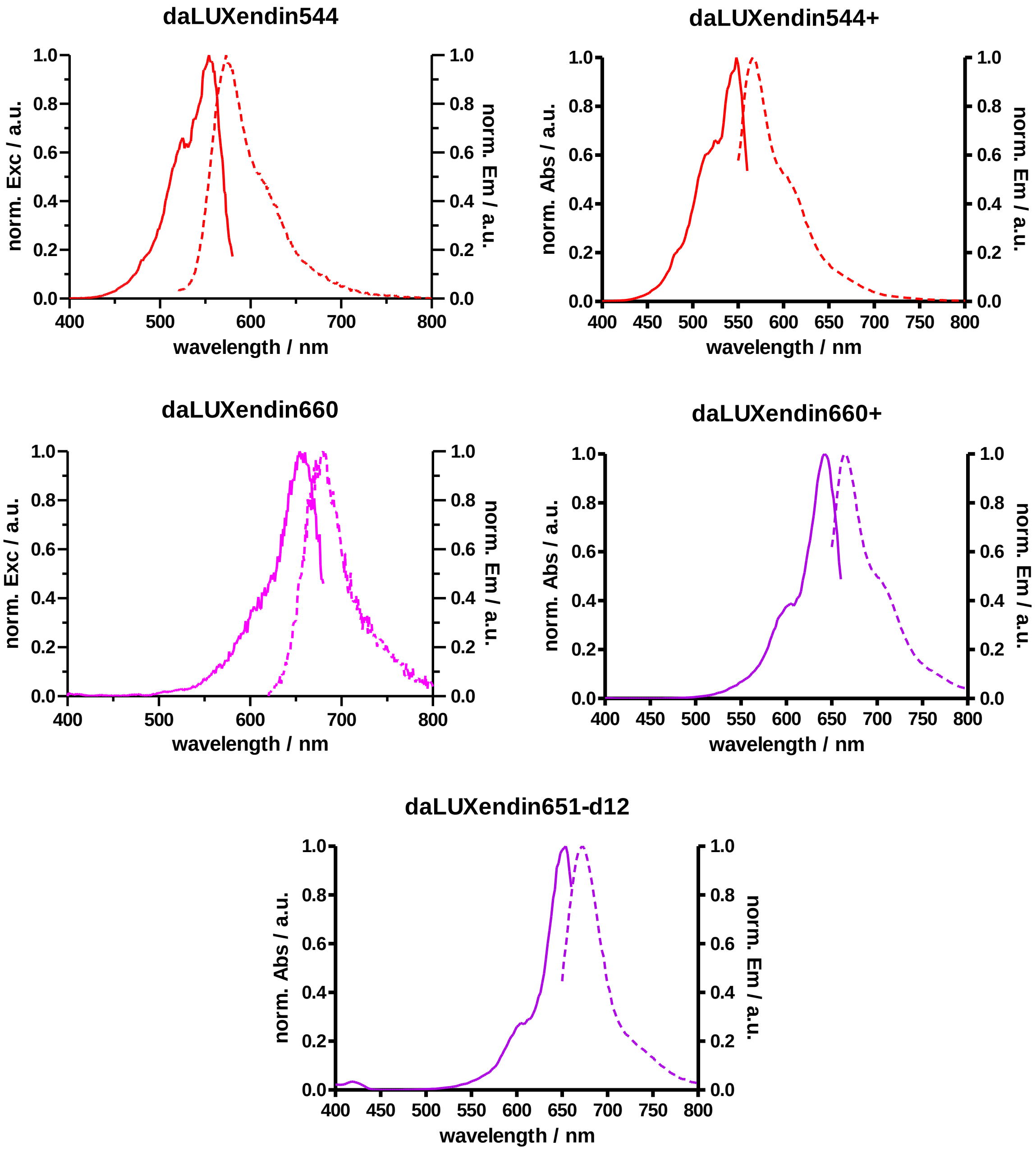

Supplementary Figure 1 Normalized fluorescence excitation and emission spectra of **daLUXendin544**, **daLUXendin544+**, **daLUXendin660**, **daLUXendin660+** and **daLUXendin651-d12**.

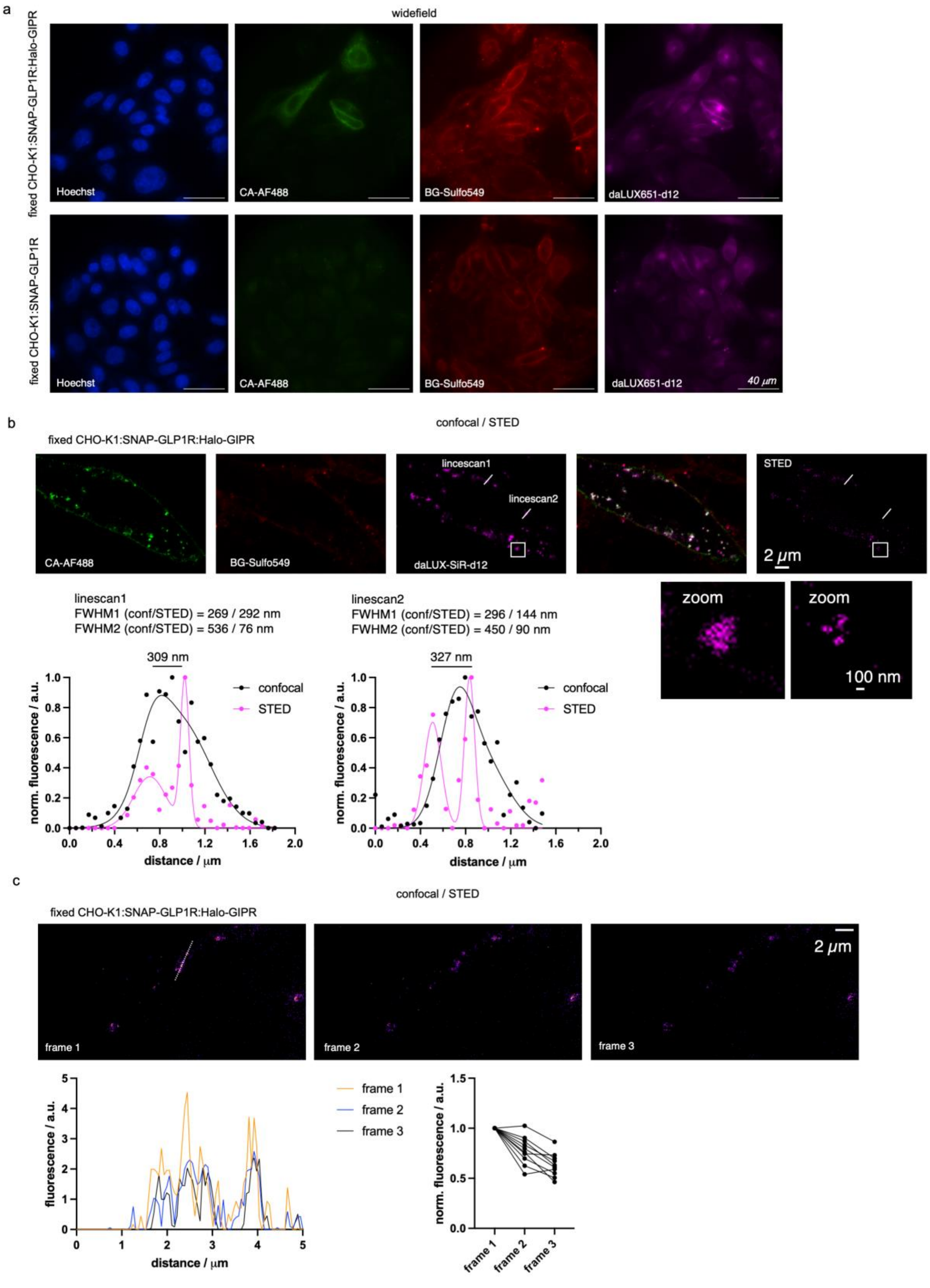

Supplementary Figure 2 Validation of daLUXendin651-d12 for cell labelling and STED imaging. a) Widefield images of fixed CHO-K1:SNAP-GLP1R:Halo-GIPR cells labelled with CA-AF488 (GIPR) and BG-Sulfo549 (GLP1R), as well as daLUXendin651-d12 (daLUX651-d12 (n = 3 wells). **b)** As for a), but showing linescan and full-width half-maximal of representative confocal and STED images (i.e. to show the improvement in resolution) (n = 8 wells). **c)** As for b), but three STED frames of daLUXendin651-d12-labelled CHO-K1:SNAP-

GLP1R:Halo-GIPR cells (n = 3 wells). Representative line scan and shows stable signal over three frames, and picked ROIs (n = 10 wells).

	hGIPR/hGIPR	mGIPR/mGLP1R
**TZP**	3.126	0.113
**daLUX544**	1.754	0.340
**daLUX660**	1.849	0.560

Supplementary Table 1 Ligand selectivity of TZP (tirzepatide), daLUX544 and daLUX660 for human GLP1R (hGLP1R), human GIPR (hGIPR), mouse GLP1R (mGLP1R) and mouse GIPR (mGIPR), calculated as fold-decrease in cAMP EC_{50} versus either GLP1 or GIP (n = 4 independent repeats).

	hGLP1R	hGIPR
**daLUX660 Kd best-fit (nM)**	2.177	8.701
**daLUX660 Kd 95% CI (nM)**	1.120 to 3.234	2.708 to 14.69
**daLUX660 Bmax best-fit (nM)**	0.0140	0.0080
**daLUX660 Bmax 95% CI (nM)**	0.0063 to 0.0217	-0.0026 to 0.0186

Supplementary Table 2 K_d and Bmax values for daLUX660 at Nluc-tagged human GLP1R (hGLP1R) and human GIPR (hGIPR) (n = 3 independent repeats).

	Ex4-Cy5		GIP-Cy5	
	GLP1R + GLP1	GLP1R + GIP	GIPR + GLP1	GIPR + GIP
**Ki (M)**	3.676e-08	N.D.	N.D.	2.085-08
	daLUX660			
	GLP1R + GLP1	GLP1R + GIP	GIPR + GLP1	GIPR + GIP
**Ki (M)**	2.727-08	N.D.	N.D.	2.724e-08

Supplementary Table 3 K_i values for hGLP1R + GLP1, hGLP1R + GIP, hGIPR + GLP1 and hGIPR + GIP determined using Ex4-Cy5, GIP-Cy5, or daLUX660 as the fluorescent probe (n = 3 independent repeats).

	hGLP1R	hGIPR
**daLUX660+ Kd best-fit (nM)**	1.375	6.458
**daLUX660+ Kd 95% CI (nM)**	-0.3582 to 3.108	1.014 to 11.90
**daLUX660+ Bmax best-fit (nM)**	0.03361	0.02314
**daLUX660+ Bmax 95% CI (nM)**	0.02597 to 0.04125	0.01887 to 0.02741

Supplementary Table 4 Kd and Bmax values for daLUX660+ at Nluc-tagged human GLP1R (hGLP1R) and human GIPR (hGIPR) (n = 3 independent repeats).

	Target	Origin	Working concentration	Supplier
Primary antibody	Insulin	Rabbit	1:500	Cell Signalling Cat# 3014S
	Glucagon	Mouse	1:2000	Sigma Cat# G2654
	GLP1R	Mouse	1:30	Developmental Studies Hybridoma Bank Cat# Mab7F38
	Somatostatin	Mouse	1:5000	Invitrogen eBioscience Cat# 14-9751-80
	Vimentin	Chicken	1:750	Abcam Cat# Ab24525
Secondary antibody	Mouse Alexa488	Goat	1:1000	Life Technologies Cat# A11001
	Mouse Alexa488	Goat	1:1000	Life Technologies Cat# A11029
	Rabbit DyLight488	Donkey	1:1000	Thermo Fisher Scientific Cat# SA5-10038
	Mouse Alexa568	Goat	1:1000	Thermo Fisher Scientific Cat# A11004
	Mouse DyLight633	Goat	1:1000	Thermo Fisher Scientific Cat# 35513
	Donkey Alexa Fluor 488,	Donkey	1:500	Thermo Fisher Scientific Cat# A78948

Supplementary Table 5 Primary and secondary antibodies

Supplementary Note 1: Chemical synthesis of fluorescent probes

Synthesis of S39C-Tirzepatide

TZP (-lipid)_S39C
Chemical Formula: $C_{188}H_{283}N_{45}O_{55}S$
M = 4085.7 g.mol^{-1}

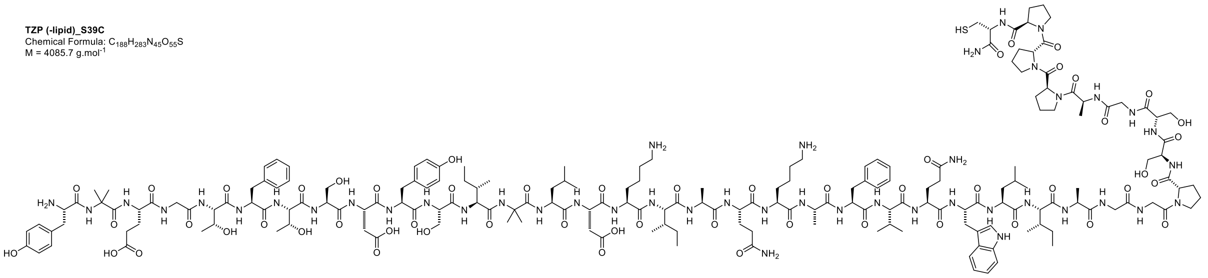

TZP_S39C
Chemical Formula: $C_{225}H_{348}N_{48}O_{67}S$
M = 4829.6 g.mol^{-1}

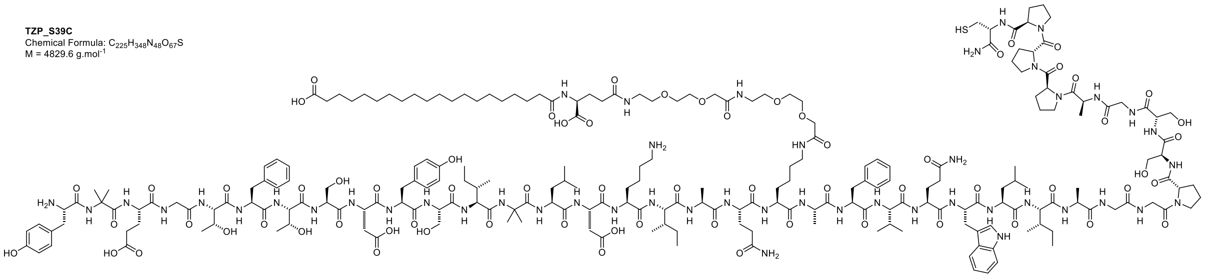

The peptides were synthesized in a 0.05 mmol scale on a Rink amide resin with a loading of 0.78 mmol/g. The synthesis was carried out on a PTI synthesizer with single couplings of each amino acid (5 eq. amino acid, 5 eq. HCTU, 5eq. Oxyma, 10 eq. DIPEA for 40 min) in DMF. The last amino acid was coupled with a Boc-protected *N*-terminus. Finally, peptides were cleaved from the resin by treatment with 2 ml of a TFA/TIS/H_2O (95:2.5:2.5) mixture for 1 h and precipitated in cold Et_2O. The crude peptides were purified by semi-preparative HPLC. The combined purified fractions were lyophilized and the **S39C-Tirzepatide** peptides were obtained as a white TFA salt.

daLUXendin544

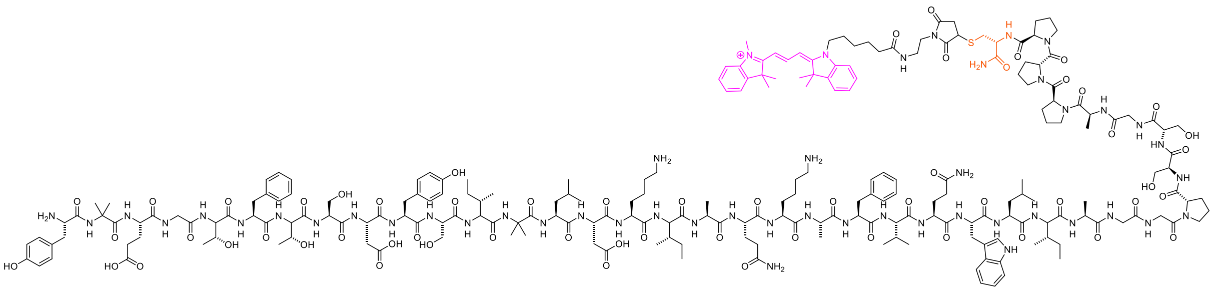

To a fresh solution of TZP(-lipid)_S39C (2.0 mg, 490 nmol, 1.0 equiv.) in PBS (400 µL) was added Cy3-maleimide (0.59 mg, 980 nmol, 2.0 equiv.) in MeCN (400 µL). The reaction mixture was incubated at 38 °C over night with gentle shaking before being subjected to RP-HPLC purification (water/MeCN gradient, 90/10 → 10/90 in 60 min). The purified fractions were combined, the concentration was determined *via* the absorption of the fluorophore at 554 nm and stocks of 5 nmol were prepared and lyophilized to yield **daLUXendin544** (255 nmol, 52%) as TFA salt. Storage was performed at -20 °C.

HRMS (QToF): calc. for $C_{224}H_{329}N_{49}O_{58}S$ $[M+3H]^{4+}$: 1166.8517, found: 1166.8503.

***t*R:** (QToF; MeCN/H_2O/formic acid = 5/95/0.1 to 95/5/0.1 over 10 min) = 4.69 min.

Fluorescence: (λ_{ex} = 544 nm, λ_{em} = 573 nm)

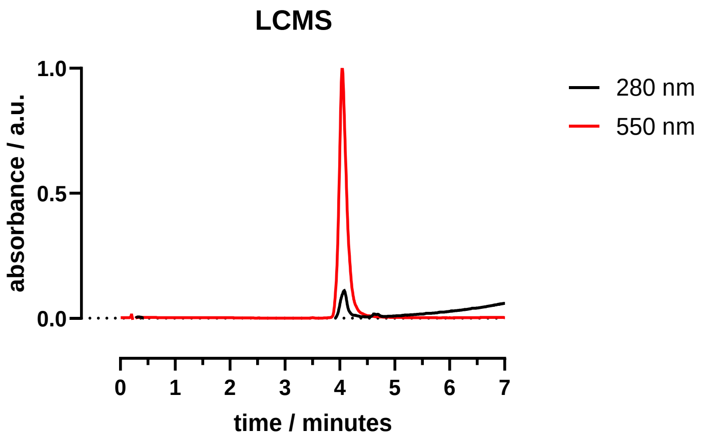

daLUXendin544+

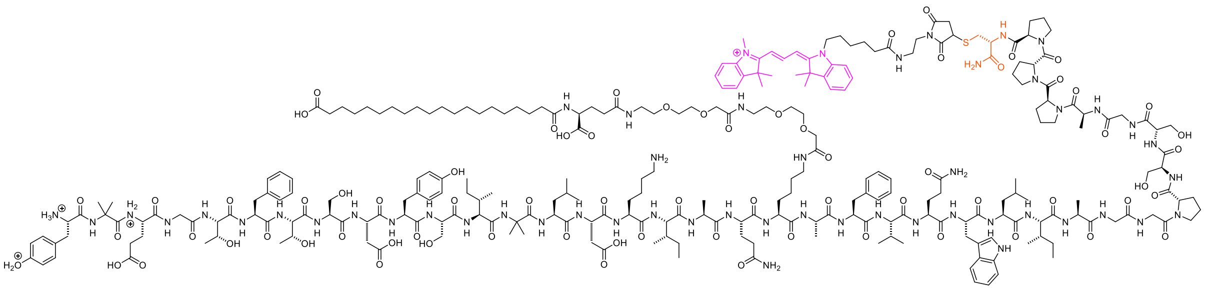

To a fresh solution of TZP_S39C (2.0 mg, 490 nmol, 1.0 equiv.) in PBS (400 µL) was added Cy3-maleimide (0.59 mg, 980 nmol, 2.0 equiv.) in MeCN (400 µL). The reaction mixture was incubated at 38 °C overnight with gentle shaking before being subjected to RP-HPLC purification (water/MeCN gradient, 90/10 → 10/90 in 60 min). The purified fractions were combined, the concentration was determined *via* the absorption of the fluorophore at 554 nm and stocks of 5 nmol were prepared and lyophilized to yield **daLUXendin544+** (94 nmol, 20%) as TFA salt. Storage was performed at -20 °C.

HRMS (QToF): calc. for $C_{261}H_{394}N_{52}O_{70}S$ $[M+3H]^{4+}$: 1352.7159, found: 1352.7794.

t_R**:** (QToF; MeCN/H_2O/formic acid = 5/95/0.1 to 95/5/0.1 over 5 min) = 3.59 min.

Fluorescence: (λ_{ex} = 548 nm, λ_{em} = 566 nm)

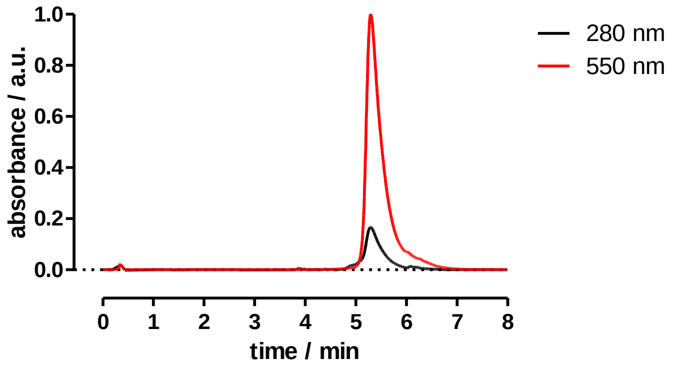

daLUXendin660

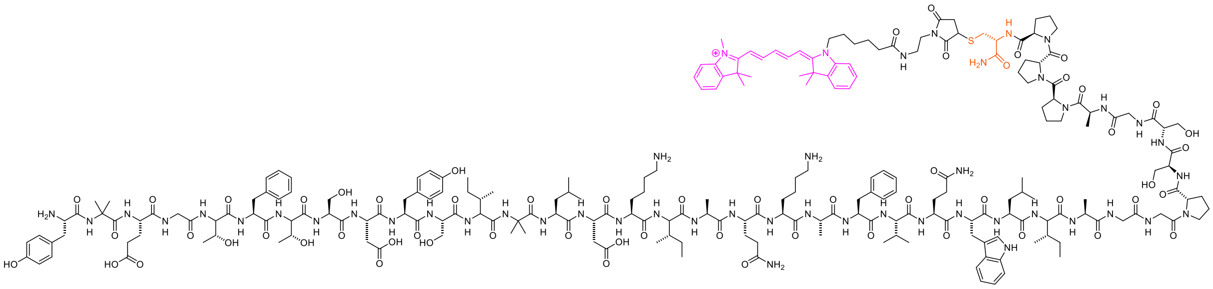

To a fresh solution of TZP(-lipid)_S39C (2.0 mg, 490 nmol, 1.0 equiv.) in PBS (400 µL) was added Cy5-maleimide (0.59 mg, 980 nmol, 2.0 equiv.) in MeCN (400 µL). The reaction mixture was incubated at 38 °C over night with gentle shaking before being subjected to RP-HPLC purification (water/MeCN gradient, 90/10 → 10/90 in 60 min). The purified fractions were combined, the concentration was determined *via* the absorption of the fluorophore at 649 nm and stocks of 5 nmol were prepared and lyophilized to yield **daLUXendin660** (230 nmol, 47%) as TFA salt. Storage was performed at -20 °C.

HRMS (QToF): calc. for $C_{226}H_{331}N_{49}O_{58}S$ $[M+3H]^{4+}$: 1173.3556, found: 1173.5955.

***t*R:** (QToF; MeCN/H_2O/formic acid = 5/95/0.1 to 95/5/0.1 over 10 min) = 4.74 min.

Fluorescence: (λ_{ex} = 660 nm, λ_{em} = 681 nm)

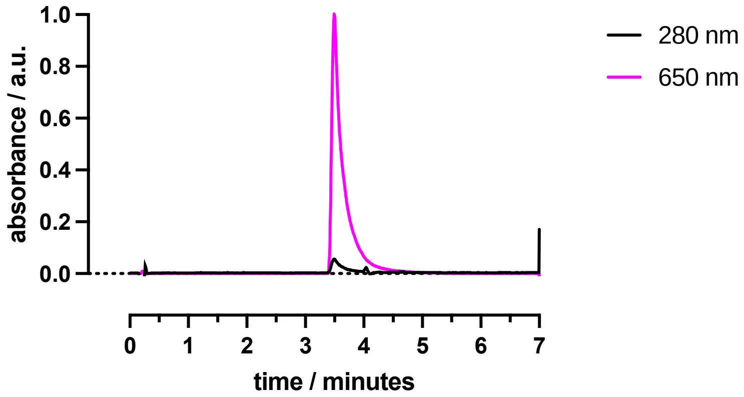

daLUXendin660+

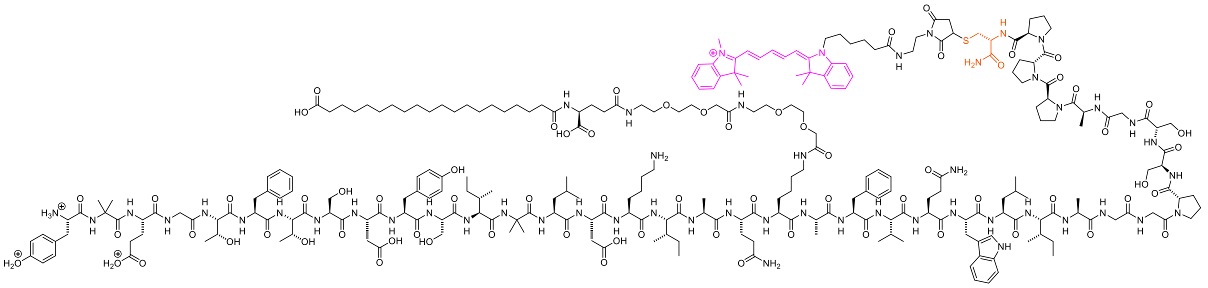

To a fresh solution of TZP_S39C (2.0 mg, 490 nmol, 1.0 equiv.) in PBS (400 µL) was added Cy5-maleimide (0.59 mg, 980 nmol, 2.0 equiv.) in MeCN (400 µL). The reaction mixture was incubated at 38 °C over night with gentle shaking before being subjected to RP-HPLC purification (water/MeCN gradient, 90/10 → 10/90 in 60 min). The purified fractions were combined, the concentration was determined *via* the absorption of the fluorophore at 649 nm and stocks of 5 nmol were prepared and lyophilized to yield **daLUXendin660+** (84 nmol, 17%) as TFA salt. Storage was performed at -20 °C.

HRMS (QToF): calc. for $C_{263}H_{396}N_{52}O_{70}S$ $[M+3H]^{4+}$: 1359.2198, found: 1359.2697.

$\boldsymbol{t_R}$: (QToF; MeCN/H_2O/formic acid = 5/95/0.1 to 95/5/0.1 over 5 min) = 3.59 min.

Fluorescence: (λ_{ex} = 642 nm, λ_{em} = 664 nm)

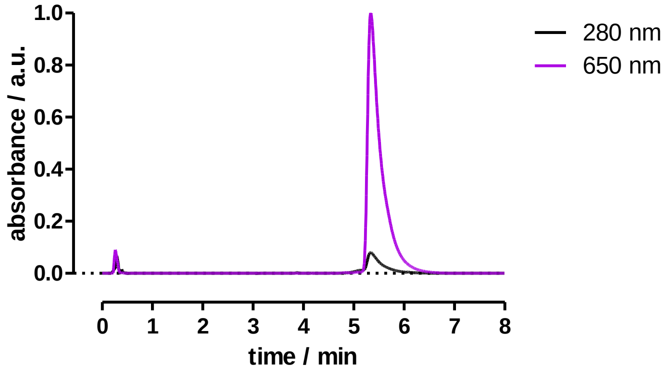

daLUXendin651-d12

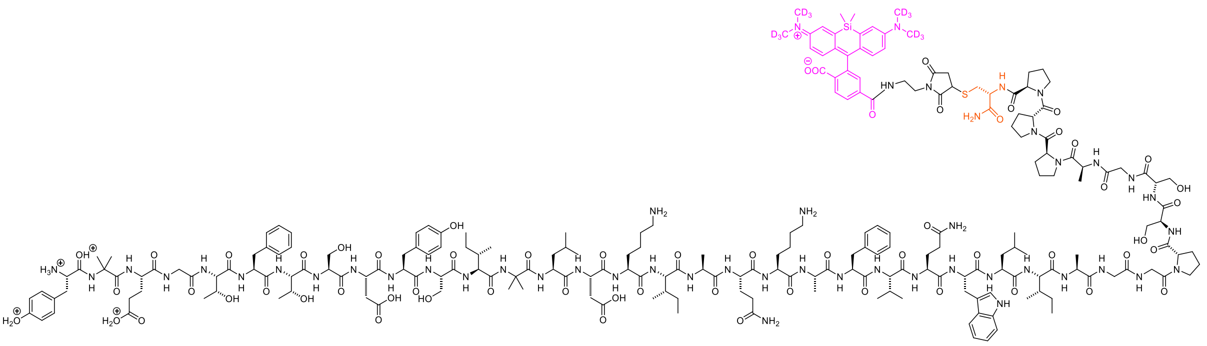

To a fresh solution of S39C-Tirzepatide (0.34 mg, 83 nmol, 1.0 equiv.) in PBS (400 µL) was added SiR-d12-maleimide (100 nmol, 1.2 equiv.) in MeCN (400 µL). The reaction mixture was incubated at 38 °C overnight with gentle shaking before being subjected to RP-HPLC purification (water/MeCN gradient, 90/10 → 10/90 in 60 min). The purified fractions were combined, the concentration was determined *via* the absorption of the fluorophore at 649 nm and stocks of 5 nmol were prepared and lyophilized to yield **daLUXendin651-d12** (25 nmol, 30%) as TFA salt. Storage was performed at -20 °C.

HRMS (QToF): calc. for $C_{221}H_{309}D_{12}N_{49}O_{60}SSi$ $[M+3H]^{4+}$: 1173.8466, found: 1173.8820.

***t*$_R$:** (QToF; MeCN/H_2O/formic acid = 5/95/0.1 to 95/5/0.1 over 5 min) = 4.53 min.

Fluorescence: (λ_{ex} = 664 nm, λ_{em} = 672 nm)

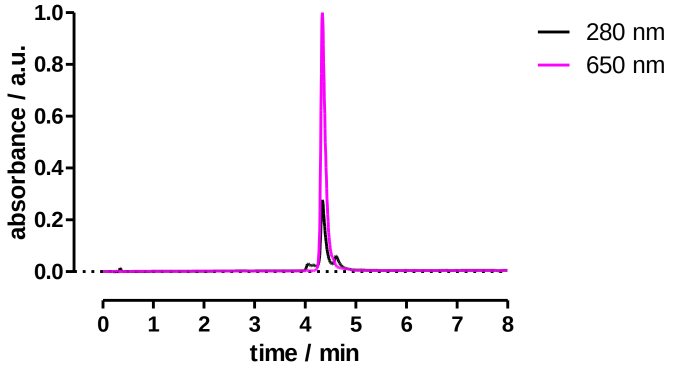

Supplement: Supplementary file 1 — Supplementary Figs. 1 and 2, Supplementary Tables 1–5, Supplementary Note 1 [file 42255_2025_1342_MOESM1_ESM.pdf]
