# Supplementary material for: Fluorescent GLP1R/GIPR dual agonist probes reveal cell targets in the pancreas and brain
Source: Nat Metab. 2025 Aug 19;7(8):1536–49. doi: 10.1038/s42255-025-01342-6 (PMC12373499; doi:10.1038/s42255-025-01342-6)
Supplement: Supplementary file 2 — Reporting Summary [file 42255_2025_1342_MOESM2_ESM.pdf]

nature portfolio

Corresponding author(s): Johannes Broichhagen, David J. Hodson

Last updated by author(s): May 12, 2025

Reporting Summary

Nature Portfolio wishes to improve the reproducibility of the work that we publish. This form provides structure for consistency and transparency in reporting. For further information on Nature Portfolio policies, see our Editorial Policies and the Editorial Policy Checklist.

Statistics

For all statistical analyses, confirm that the following items are present in the figure legend, table legend, main text, or Methods section.

n/a	Confirmed	
☐	☒	The exact sample size (*n*) for each experimental group/condition, given as a discrete number and unit of measurement
☐	☒	A statement on whether measurements were taken from distinct samples or whether the same sample was measured repeatedly
☐	☒	The statistical test(s) used AND whether they are one- or two-sided<br>*Only common tests should be described solely by name; describe more complex techniques in the Methods section.*
☒	☐	A description of all covariates tested
☐	☒	A description of any assumptions or corrections, such as tests of normality and adjustment for multiple comparisons
☐	☒	A full description of the statistical parameters including central tendency (e.g. means) or other basic estimates (e.g. regression coefficient) AND variation (e.g. standard deviation) or associated estimates of uncertainty (e.g. confidence intervals)
☐	☒	For null hypothesis testing, the test statistic (e.g. *F*, *t*, *r*) with confidence intervals, effect sizes, degrees of freedom and *P* value noted<br>*Give P values as exact values whenever suitable.*
☒	☐	For Bayesian analysis, information on the choice of priors and Markov chain Monte Carlo settings
☒	☐	For hierarchical and complex designs, identification of the appropriate level for tests and full reporting of outcomes
☒	☐	Estimates of effect sizes (e.g. Cohen's *d*, Pearson's *r*), indicating how they were calculated

Our web collection on statistics for biologists contains articles on many of the points above.

Software and code

Policy information about availability of computer code

Data collection	Images were acquired using Zen 2012 (Zeiss), Olympus cellSens and STEDYCON 9.0.799-g22f03ed2 software.<br><br>Localization coordinates were acquired using Abbelight NEO software.
Data analysis	Images were analyzed using ImageJ 1.5j8 (NIH), Zen 3.5 (Blue Edition; Zeiss), Olympus cellSens v3.2, Abbelight NEO v39 or STEDYCON 9.0.799-g22f03ed2 software. Numerical data were analyzed using R Project 4.4.3 and Prism 8, 9 or 10 (Graphpad).<br><br>Particle clustering was analyzed using DBSCAN, implemented in Abbelight NEO or the DBSCAN R Project package (https://cran.r-project.org/web/packages/dbscan/index.html). Single particle tracking was performed using Trackmate plugin for ImageJ.

For manuscripts utilizing custom algorithms or software that are central to the research but not yet described in published literature, software must be made available to editors and reviewers. We strongly encourage code deposition in a community repository (e.g. GitHub). See the Nature Portfolio guidelines for submitting code & software for further information.

Data

Policy information about availability of data

All manuscripts must include a data availability statement. This statement should provide the following information, where applicable:

- Accession codes, unique identifiers, or web links for publicly available datasets
- A description of any restrictions on data availability
- For clinical datasets or third party data, please ensure that the statement adheres to our policy

Source data are provided with this paper. Other datasets generated and/or analyzed during the current study are available from the corresponding authors upon request. Due to their large size, individual raw image files are available upon request. upon request from J.B. or D.J.H., who will respond within 30 working days. daLUXendins are subject to a Material Transfer Agreement and ability to manufacture and supply. All requests for reagent and data will be handled by J.B. or D.J.H., who will respond within 30 working days.

Human research participants

Policy information about studies involving human research participants and Sex and Gender in Research.

Reporting on sex and gender	N/A
Population characteristics	N/A
Recruitment	N/A
Ethics oversight	N/A

Note that full information on the approval of the study protocol must also be provided in the manuscript.

Field-specific reporting

Please select the one below that is the best fit for your research. If you are not sure, read the appropriate sections before making your selection.

☒ Life sciences ☐ Behavioural & social sciences ☐ Ecological, evolutionary & environmental sciences

For a reference copy of the document with all sections, see nature.com/documents/nr-reporting-summary-flat.pdf

Life sciences study design

All studies must disclose on these points even when the disclosure is negative.

Sample size	The measurement unit is animal, batch of islets, batch of iPSC-derived islets, or well of cells. Experiments were repeated independently at least twice, usually with technical replicates. Islet data are reported from at least three separate isolation procedures. iPSC-derived islet data are reported from at least two separate differentiations. Effect size was determined from pilot studies as well as similar experiments in the published literature. A priori sample size calculations were performed in G*Power 3.1.9.2 based upon the expected effect size (d), using a test power = 0.9, alpha = 0.05 and the difference between two independent means.
Data exclusions	No data were excluded unless the cells displayed a clear non-physiological state (i.e. impaired viability) and/or positive and negative controls failed.
Replication	All findings were replicated across multiple cells, islets and animals, with independent repeats.
Randomization	Samples and animals were allocated to treatment groups in a randomized manner to ensure that all states were represented in the different experiment arms.
Blinding	Data were acquired using imaging setups that performed the measurement independently of the observer.

Reporting for specific materials, systems and methods

We require information from authors about some types of materials, experimental systems and methods used in many studies. Here, indicate whether each material, system or method listed is relevant to your study. If you are not sure if a list item applies to your research, read the appropriate section before selecting a response.

Materials & experimental systems

n/a	Involved in the study	
☐	☒	Antibodies
☐	☒	Eukaryotic cell lines
☒	☐	Palaeontology and archaeology
☐	☒	Animals and other organisms
☒	☐	Clinical data
☒	☐	Dual use research of concern

Methods

n/a	Involved in the study	
☒	☐	ChIP-seq
☒	☐	Flow cytometry
☒	☐	MRI-based neuroimaging

Antibodies

Antibodies used

INS Cell Signaling Technology Cell Signaling Technology Cat# 3014, RRID:AB_2126503.
GCG Sigma-Aldrich Sigma-Aldrich Cat# G2654, RRID:AB_259852.
SST Thermo Fisher Scientific Thermo Fisher Scientific Cat# 14-9751-80, RRID:AB_2572981.
GLP1R Developmental Studies Hybridoma Bank #Mab7F38, RRID:AB_2618101.
Chicken anti-vimentin Abcam Cat# Ab24525, RRID:AB_778824.
DyLight 488 Thermo Fisher Scientific Thermo Fisher Scientific Cat# SA5-10038, RRID:AB_2556618.
Alexa 488 Thermo Fisher Scientific Thermo Fisher Scientific Cat# A-11001, RRID:AB_2534069
Alexa 488 Thermo Fisher Scientific Thermo Fisher Scientific Cat# A-11029, RRID:AB_2534088
Alexa 488 Thermo Fisher Scientific Thermo Fisher Scientific Cat# A-78948, RRID:AB_2921070
Alexa 633 Thermo Fisher Scientific Thermo Fisher Scientific Cat# A-21052, RRID:AB_2535719
DyLight 633 Thermo Fisher Scientific Thermo Fisher Scientific Cat# 35513, RRID:AB_1965952
Alexa 568 Thermo Fisher Scientific Thermo Fisher Scientific Cat# A10042, RRID:AB_2534017

Dilutions are listed in Supplementary Table 5.

Validation

All antibodies have been reported to be specific, based upon one or more of the following criteria: 1) cell-type specific localization; 2) absence of staining following pre-adsorption with antigen; 3) absence of staining in knockout tissue; or 4) absence of staining in cells non-transfected with cDNA encoding protein target.

INS Cell Signaling Technology Cell Signaling Technology Cat# 3014, RRID:AB_2126503. Antibody was validated in islets lacking INS.
GCG Sigma-Aldrich Sigma-Aldrich Cat# G2654, RRID:AB_259852. Antibody was validated in PCSK2-/- mice that lack GCG.
SST Thermo Fisher Scientific Thermo Fisher Scientific Cat# 14-9751-80, RRID:AB_2572981. Antibody was validated in tissue from SST-/- mice.
GLP1R Developmental Studies Hybridoma Bank #Mab7F38, RRID:AB_2618101. Antibody was validated in tissue from GLP1R-/- mice.
Chicken anti-vimentin Abcam Cat# Ab24525, RRID:AB_778824. Validated using western blot as well as specific localization to astrocytes and vascular endothelial cells.
DyLight 488 Thermo Fisher Scientific Thermo Fisher Scientific Cat# SA5-10038, RRID:AB_2556618. Antibody was validated in mice that lack SST.

Eukaryotic cell lines

Policy information about cell lines and Sex and Gender in Research

Cell line source(s)

AD293 (Agilent, RRID:CVCL_9804).

HEK293T cells (European Collection of Authenticated Cell Cultures, RRID:CVCL_0063).

Human induced pluripotent stem cells (iPSC) (ALSTEM #iPS11).

CHO-K1 (European Collection of Authenticated Cell Cultures, RRID:CVCL_0214).

MIN6-CB4 (a kind gift from Dr Jun-Ichi Miyazaki and Dr Satsuki Miyazaki, Osaka University).

Authentication

AD293 cells were authenticated using STR profiling.

HEK293T cells were authenticated using STR profiling.

ALSTEM #iPS11 cells are authenticated at source by testing for expression of OCT4 and TRA-1-60, as well as alkaline phosphatase activity.

CHO-K1 cells were authenticated at source using DNA barcoding and DNA profiling.

MIN6-CB4 cells were authenticated using GLP1R labelling (beta cell specific marker), as well as insulin staining and insulin secretion.

Mycoplasma contamination

All cell lines tested negative for mycoplasma, with testing performed every 3 months.

Commonly misidentified lines (See ICLAC register)	N/A

Animals and other research organisms

Policy information about studies involving animals; ARRIVE guidelines recommended for reporting animal research, and Sex and Gender in Research

Laboratory animals	GLP1R-SNAP/SNAP knock-in mice, generated using CRISPR on a C57BL/6J background,, were used for islet labelling studies. Homozygous and wild-type littermates were used in a number of experiments.<br><br>GLP1RKO/KO mice, generated using CRISPR on a C57BL/6J background, were used for islet labelling studies. Homozygous (GLP1RKO/KO), heterozygous (GLP1RWT/KO) and wild-type (GLP1RWT/WT) littermates were used in a number of experiments.<br><br>GLP1R-/- mice, generated using CRISPR on a C57BL/6J background, were used for IPGTT studies. Homozygous, heterozygous and wild-type littermates were used in a number of experiments.<br><br>C57BL6/N mice were used for food intake studies.<br><br>GLP1R-Cre:tdRFP mice, GIPR-Cre:GFP and GIPR-Cre:GCaMP6 reporter animals (all on a (both on a C57BL/N background) were used for brain labelling studies.<br><br>CD1 and C57BL6/J mice were used as tissue donors for islet labelling studies.<br><br>All animals were male and female, 6-14 wks. For glucose tolerance testing, mice were socially-housed in specific-pathogen free conditions under a 12 h light-dark cycle with ad libitum access to food and water, relative humidity 55±10% and temperature 21±2° C. For food intake studies, mice were kept on a 12 h light/dark cycle at 20–24°C and 45–65% relative humidity (typically 21°C and 55%).<br><br>All genetically-modified mouse lines have been previously well-phenotyped and reported.
Wild animals	No wild animals were used in the studies.
Reporting on sex	All studies combined males and females, since: 1) no sex-specific phenotype has been reported for any of the genetically-modified lines used; and 2) male and female tissues were indistinguishable by their chemical probe or SNAP-tag labelling.<br><br>Studies are insufficiently powered to disaggregate according to sex, but neither require disaggregation due to absence of phenotypic and/or labelling differences between the sexes.
Field-collected samples	N/A
Ethics oversight	Animal studies were regulated by the Animals (Scientific Procedures) Act 1986 of the U.K. (Personal Project Licences P2ABC3A83, PP1778740 and PP6526002). Approval was granted by the University of Birmingham, University of Oxford and UCL Animal Welfare and Ethical Review Bodies (AWERB).

Note that full information on the approval of the study protocol must also be provided in the manuscript.
